# Low-cost and scalable machine learning model for identifying children and adolescents with poor oral health using survey data: An empirical study in Portugal

**DOI:** 10.1371/journal.pone.0312075

**Published:** 2025-01-24

**Authors:** Susana Lavado, Eduardo Costa, Niclas F. Sturm, Johannes S. Tafferner, Octávio Rodrigues, Pedro Pita Barros, Leid Zejnilovic

**Affiliations:** 1 Nova School of Business and Economics, Universidade Nova de Lisboa, Carcavelos, Portugal; 2 CEGIST - Centre for Management Studies, Instituto Superior Técnico, Universidade de Lisboa, Carcavelos, Portugal; 3 Nova School of Information Management, Universidade Nova de Lisboa, Carcavelos, Portugal; 4 Associação Portuguesa Promotora de Saúde e Higiene Oral, Seixal, Portugal; Danube Private University, AUSTRIA

## Abstract

This empirical study assessed the potential of developing a machine-learning model to identify children and adolescents with poor oral health using only self-reported survey data. Such a model could enable scalable and cost-effective screening and targeted interventions, optimizing limited resources to improve oral health outcomes. To train and test the model, we used data from 2,133 students attending schools in a Portuguese municipality. Poor oral health (the dependent variable) was defined as having a Decayed, Missing, and Filled Teeth index for deciduous teeth (dmft) or permanent teeth (DMFT) above expert-defined thresholds (dmft/DMFT ≥ 3 or 4). The survey provided information about the students’ oral health habits, knowledge, beliefs, and food and physical activity habits, which served as independent variables. Logistic regression models with variables selected through low-variance filtering and recursive feature elimination outperformed various others trained with complex machine learning algorithms based on precision@k metric, outperforming also random selection and expert rule-based models in identifying students with poor oral health. The proposed models are inherently explainable, broadly applicable, which given the context, could compensate their lower performance (Area Under the Curve = 0.64—0.70) compared to similar approaches and models. This study is one of the few in oral health care that includes bias auditing of classification models. The audit surfaced potential biases related to demographic factors such as age and social assistance status. Addressing these biases without significantly compromising model performance remains a challenge. The results confirm the feasibility of survey-based machine learning models for identifying individuals with poor oral health, but further validation of this approach and pilot testing in field trials are necessary.

## Introduction

Dental caries, a common yet preventable chronic disease, is a significant health concern among children, particularly those from disadvantaged backgrounds [1–4]. Addressing caries and enhancing children’s oral health necessitates not only expanding the capacity of dental services but also implementing strategies to increase the utilization of existing services [2]. Caries-risk assessment tools are important for developing such strategies [5, 6]. These tools help the facilitation of targeted interventions, optimization of resource allocation, and promotion of equitable access to preventive care [6]. They are particularly interesting when they afford scalability and cost-efficiency, like in the case of a screening tool that uses self-reported data [7–9]. Although machine learning started gaining prominence in the oral health academic literature for both assessing the risk of new caries [5, 10] and screening to identify the current status of caries [6, 9, 11], the evidence of the performance of machine learning models for caries prediction or identification is still limited. With this study, we attempted to contribute to that body of evidence using comprehensive self-reported and clinical data of school-attending children and adolescents aged 5 to 19.

The motivation for this study was the need identified by a social organization, Associação Portuguesa Promotora de Saúde e Higine Oral (APPSHO), to develop a scalable and cost-effective algorithmic model, a tool that identifies students with poor oral health to better direct resources toward them, incentivizing visit to dentists, and promoting better behaviors and oral health outcomes. In Portugal, there is a government program that offers vouchers to children for free dental visits, although the utilization of these vouchers is suboptimal [12]. The existence of the requested model would help identify who to incentivize more and how, and potentially improve the impact of public funds investment for better oral health. We defined that a student has poor oral health if they have the index of decayed, missing, and filled teeth (deciduous teeth—dmft; or permanent teeth—DMFT) higher than a threshold value established by a panel of dentists. The rationale is that students with so-defined poor oral health would require relatively urgent intervention from oral health professionals. The proposed approach would serve as an alternative to in-person screening for which, although expensive, there is no sufficient evidence of significant impact on oral health outcomes [13].

There are several assessment tools in dentistry that, at least partially, fill the need to identify students with poor oral health. The Caries Management Risk Assessment (CAMBRA) [14], which was used to assess caries risk at a university instructional clinic, or the Cariogram [15–18] are good examples of such tools which exhibited moderate to good results in practice. However, these risk-assessment systems require using data obtained through specialized clinical observations, such as past caries experience and salivary and microbiological variables, besides sociodemographic and behavioral variables [17]. In recent years, there has been an increased interest in using machine learning (ML) and extensive datasets to estimate individual caries risk. Pang and colleagues [5] have used a random forest algorithm to train a model for predicting teenagers’ risk of developing additional caries based on demographic, socioeconomic, behavioral, and genetic factors as well as past caries experience, achieving an Area Under the Curve (AUC) of 0.74. Other researchers proposed a model trained by a multi-modal deep neural network to estimate the presence of caries based on a comprehensive dataset including survey and clinically collected dental image data with a precision of 0.89 [19]. Previous research has also leveraged demographic and observational data derived from medical consultation, including specific parameters such as infectious tooth parameters collected by experts (although the authors did not specify the variables used), to develop a model using a random forest algorithm to identify caries in 12-year-old children with a precision of 0.94 [20]. Karhade and colleagues [6] developed an auto-machine-learning model as a screening tool for preschool children to identify their early childhood caries status, achieving relatively high specificity with only survey data and even extremely parsimonious models.

While the results of these models are very encouraging, showing the ability of ML techniques to identify students at risk of poor oral health, in many cases, they require large and extensive datasets and depend on the collection of invasive data, which are costly to obtain due to the required time, the use of specialized environment, and significant human resources. Although very effective, because of their complexity and cost, large-scale application of such models in large cohorts of children and adolescents in schools is very difficult, limiting their usability at a national level. There are studies that only rely upon survey data to build ML models for dmft/DMFT prediction or screening, but still too few, and usually with target populations limited to specific segments of either children or adolescents and rarely to both, with a few exceptions [21, 22].

In this study, we support the argument that contrary to the approaches of feeding models with extensive and invasive data, models that favor usability and scalability may be more applicable in some settings despite the potential loss of accuracy. There are successful examples of resolving this trade-off and achieving scalability and performance. For example, Hung and colleagues [8] surveyed and examined adults in the United States, creating a large data set that was used to train a model with 37 self-reported variables. They identify the presence or absence of root caries using a model trained by the Support Vector Machine algorithm, with a reported accuracy of 97% and a precision of 95% [8]. This approach can be easily scaled for massive screening work, which is especially interesting for public health interventions and screening of oral health among children and young adolescents.

The objective of the study is to test whether it is feasible to develop and evaluate a machine learning model to identify students (5 to 19 years old) with poor oral health using information collected through surveys. To achieve that objective, we used self-reported survey data (independent variables) matched with clinical screening data collected by APPSHO dentists (target or dependent variable) for all students in one Portuguese municipality. The proposed model would facilitate the rapid identification of students with potentially compromised oral health, offer scalability at a relatively low cost, and enable the implementation of targeted preventive interventions to improve dental well-being among children and adolescents.

We hypothesized that low-cost, survey-based models can outperform rule-based models constructed with oral experts (our baseline model) for the children and adolescents population. Hence, our null hypothesis was that there would be no statistically significant differences in the performance of our survey-based model compared with the baseline rule-based models.

## Materials and methods

### Data collection

Data collection was approved by the Portuguese National Data Commission (Comissão Nacional de Proteção de Dados), process number 14108/2011, approval number 12400/2011, from November 21, 2011. All students from the urban municipality of Seixal in the Lisbon region of Portugal were invited to participate in the study, and written consent was obtained from the students’ tutors. At the beginning of the study, personal data, such as student names and tutor contact information, were collected to facilitate the distribution of oral health reports. Upon completion of the data collection, all data were digitized, matched, and anonymized. After verification of anonymization, the original survey responses with identifiers (e.g., names) were destroyed. We had access only to anonymized historical digitized data for the 4,216 students who participated in the survey and/or oral health screenings.

This dataset comprises clinical observations collected by oral health professionals from APPSHO and survey responses reported by students, with support from their tutors. The maximum time interval between survey and clinical observation was 120 days. Data collection occurred between 2008 and 2015, with nearly 80% of screenings occurring in 2012 or later. Matching the survey and clinical observation data resulted in 2,133 paired observations.

The oral health of the students was assessed using the decayed, missing and filled teeth index for permanent teeth (DMFT) and deciduous teeth (dmft). These indices are the predominant measure of the population’s exposure to caries worldwide, particularly in children [23–27]. Additionally, they are frequently employed in studies evaluating caries risk assessment systems [17, 28]. The indices were calculated following the World Health Organization (WHO) guidelines [29].

In addition to the dmft and DMFT indices, the dataset included student survey data with information about three general topics: oral health habits and beliefs (including oral hygiene practices, dental visits, and oral health knowledge), nutritional habits (detailing meal frequency and food choices), and physical activity habits (capturing exercise frequency and sports participation).

### Pre-registration

This study originated as an applied interdisciplinary project: a collaboration of a data science team with a social organization to improve oral health among children. However, the decision to study the approach proposed in this study came after the data had been collected. For this reason, the study was not preregistered.

### Data pre-processing and the construction of new variables

The first steps of data processing included 1) merging survey and clinical observation data; 2) cleaning out-of-range values; 3) imputing missing values, using the average value of the population cohort; 4) performing data transformations, creating flags for categorical data and variables counting the number of food items typically ingested, aggregated in four categories. We described all the variables considered in this study in S1 and S2 Tables. The mapping of food items into categories has been carried out as shown in S3 Table.

**Fig 1:**
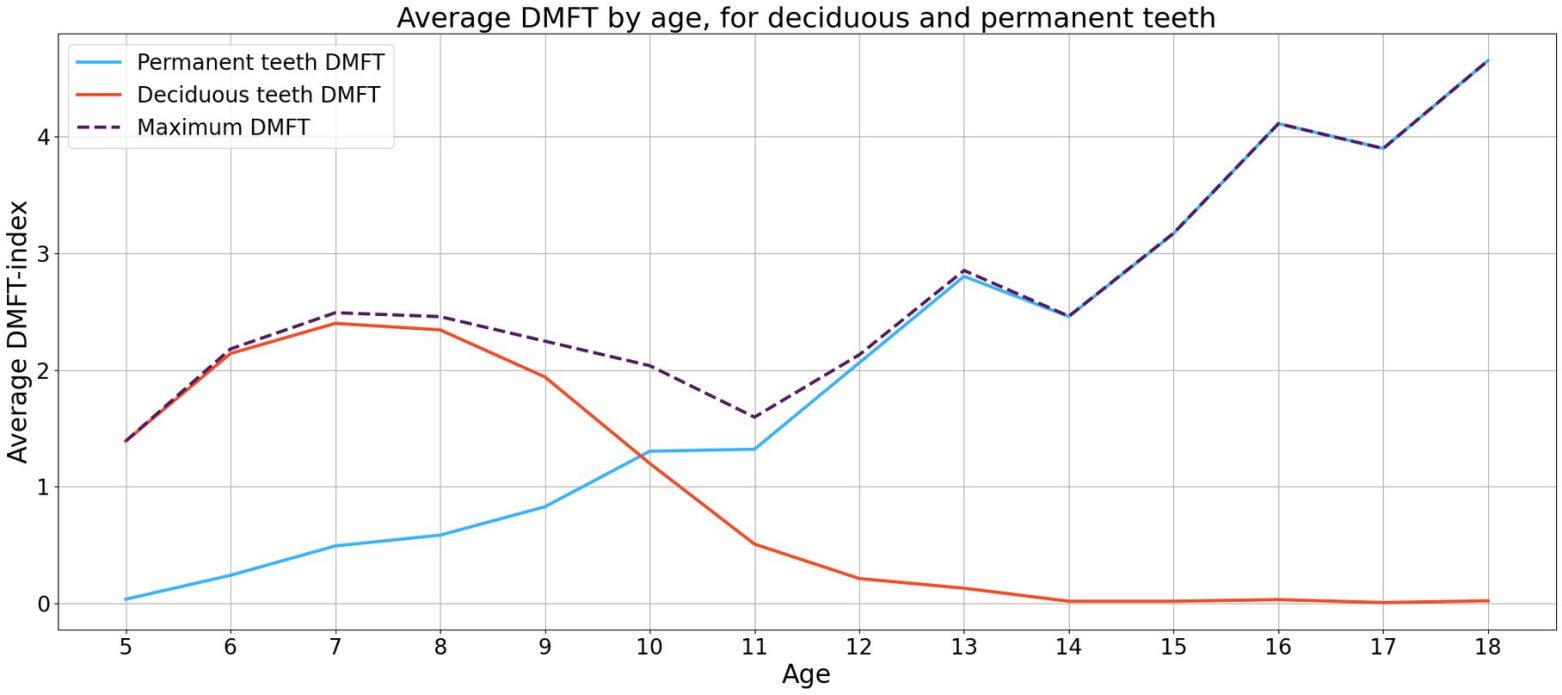
Average dmft/DMFT index by age for deciduous and permanent teeth.

Both dmft and DMFT indices were used in this study, as the dataset included young children with mixed dentition (where both indices apply) and adolescents with only permanent teeth (where only the DMFT index applies). Our target variable (what the models learned to estimate) was a binary variable, where value one (1) indicates a high risk of poor oral health, and value zero (0) indicates a low risk. To train a classification model for the entire sample, we constructed the target variable by comparing students’ dmft and DMFT indices and always selecting the higher of the two values. This approach was chosen because a) when transitioning from deciduous to permanent teeth, the DMFT values are usually lower than the dmft values, as recently erupted teeth did not have enough time to develop caries (see Fig 1); and b) literature suggests that dmft values are good predictors of DMFT values five years later [24]. In the remainder of this article, we referred to this metric as the dmft/DMFT index. To convert the dmft/DMFT index from a continuous to a binary variable, we consulted oral health experts from APPSHO, with whom we established two clinically relevant thresholds, dmft/DMFT ≥ 3 and dmft/DMFT ≥ 4. We created two target variables, one for each threshold, assigning a value of 1 if the dmft/DMFT index was higher than the threshold and 0 otherwise. This binary variable is interpreted as an indicator of a risk of poor oral health. Note that we conducted our statistical analysis and the evaluation of the two models considering the entire sample and two subsamples, created by splitting the original sample, where one subsample comprises children younger than 12 and the other of those 12 years old or older. Depending on the sample and the indices used for creating the variables, we referred to the binary target variables in the remainder of the manuscript as the binary dmft (when using a subsample of children less than 12 years old), binary DMFT (for those 12 years old or older), or binary dmft/DMFT index (when considering the entire sample and maximum dmft/DMFT).

### Statistical analysis

In the first part of the statistical analysis, we produced descriptive statistics, computing the distribution of categorical variables. Means and standard deviations (SD) of numeric variables were computed using the Python Pandas library [30], version 1.5.3. Data visualizations were produced using Python’s Matplotlib [31], version 3.7.4. Two-tailed independent sample t-tests were conducted using the SciPy Python library [32] version 1.10.1., to test differences between the mean values of the dependent variable for different groups of students. We considered the alpha level of statistical significance to be 0.05. That means that p-values (p) below 5% (*p* < 0.05) indicated statistically significant differences between different groups of students. Note that in the results section, we show means and standard deviation as mean(standard deviation).

### Model training

We trained two classification models, considering the entire sample (all ages) and binary dmft/DMFT as the target variable. One model was trained with binary dmft/DMFT for which we used a threshold level of three, and the other model with binary dmft/DMFT with a threshold level of four. Model training and evaluation were implemented using the Scikit-Learn Python library [33], version 1.1.3, a commonly used open-source Python library for machine learning. We employed many machine learning algorithms to train the classification models, including Logistic Regression, Naïve Bayes, Support Vector Machines, Decision Trees, Random Forest, XGBoost, K-Nearest Neighbors, and Neural Networks (Multi-Layer Perceptrons). For each algorithm, the best models were selected through grid search with hyperparameter optimization, also using the Scikit-Learn Python library version 1.1.3. Following previous research, we tested for agreement between the risk levels assigned by the two models to the same student using Cohen’s Kappa values [34].

We adopted the following validation strategy to ensure robust model selection. First, we reserved 20% of the dataset for final testing (hold-out test set). For model training and validation, we employed 5-fold cross-validation. This involved splitting the remaining data into five folds; one fold served as the validation set, while the others comprised the training set. We iterated this process, rotating the validation fold, to optimize hyperparameters and gauge model performance on unseen data. The final model performance was calculated by averaging results across validation folds. Subsequently, the best-performing model was retrained on the entire training set and evaluated on the held-out test set, yielding an accurate estimate of its generalization to new data.

### Model performance evaluation

The models estimated probabilities of students having poor oral health, the dmft/DMFT index higher than the predefined threshold. These probabilities were then sorted from highest to lowest, producing a ranked list that served as a basis to inform actionable decisions, such as selecting or not a student for an oral health intervention. We used the Precision@k as the metric to evaluate and select models [35]. Precision measures the proportion of students correctly identified by the model as having the dmft/DMFT index above the pre-defined threshold (poor oral health) out of all students the model flagged as having poor oral health. Precision@k represents the value of precision when a given percentage of students (k) is selected for intervention from the ranked list. The value of k may be adjusted to balance efficiency and inclusiveness constraints, considering that governments may have limited resources to intervene with students. The Precision@k was selected based on the assumption that resource-constrained assistive programs must prioritize students with poor oral health, minimizing false positives during the selection. By minimizing false positives in a fixed number of students selected for intervention, we also minimize false negatives as more students truly having poor oral health are selected. During model selection, we estimated the models’ performance for different values of k to provide the flexibility to balance the efficiency and breadth of the selected population. Typically, the larger the selected population, the fewer truly at-risk students are left out at the expense of more false positives.

To compare the performance of the proposed system against other deployment alternatives, and in the absence of an existing practice, we built two baseline models: i) a model where students would be randomly selected as having poor oral health and ii) a model based on experts’ rules to identify students with poor oral health. To create the experts’ model, we designed a survey asking a panel of eight APPSHO dentists to identify the best predictors of students’ current dmft/DMFT index. We used the average weights from the experts’ answers to generate an equation that attributed a risk score to each of the students in the dataset, which was then used to rank the students in terms of risk and calculate precision@k. Details on the factors and respective weights can be found in S1 File.

### Bias auditing

We audited our model outcomes for bias by calculating the disparities in the errors made by the classifier for different demographic groups [36, 37], when considering the top 10% of students with the higher scores attributed by the model as being more likely at high risk of poor oral health. The disparities were calculated by dividing the metric of choice of one group over the metric of another group (the reference group). Our chosen metric was the true positive rate (TPR), also known as sensitivity or recall, which is the proportion of students who have a dmft/DMFT index above the pre-defined threshold and are correctly identified by the model as such. That metric was selected because the proposed model is meant to be used in assistive interventions with limited resources, meaning that we could only intervene in a small fraction of the population. Thus, we were mainly concerned with high-risk students who might not get selected as such by the model [38]. We considered there were equitable outcomes if the TPR disparity was between 0.80 and 1.25 [38]. To conduct a bias audit, we used the Aequitas Python library (version = 0.42.0), an open-source library developed specifically for this purpose [38]. We analyzed potential bias in all demographic variables available in our dataset (age, gender, and social support). For instance, we checked whether our model systematically identified a higher proportion of girls with a dmft/DMFT higher than the threshold than boys with a dmft/DMFT higher than the threshold by measuring disparities in the groups’ True Positive Rate (TPR). Since the audit revealed some bias, we showed the impact of mitigating those biases in model performance [39].

### Analyses by age groups

The dmft and DMFT indices are significantly influenced by age as children transition from deciduous to permanent dentition. The WHO recommends using the DMFT index for adolescents aged 12 and older and the dmft index for children five or six years old or younger, but no specific guidelines are provided for those between these ages [29]. Recent studies on the oral of children aged five to 12 years have analyzed the dmft and DMFT indices separately [23, 40, 41], including epidemiological studies research in Portugal aimed at informing national public health policies [42].

Thus, in line with the common practice, in addition to analyzing descriptive statistics, t-tests, and evaluating model performance and bias for the entire population as described above, we conducted separate analyses for students aged 12 and older and for students aged five to 11. For the older group, only the DMFT index was used as the dependent variable. For the younger students, we conducted separate analyses using both the dmft and DMFT indices as dependent variables. To assess model performance and audit bias for these age groups, we used binary dmft and binary DMFT indices as described in the ‘Data pre-processing and the construction of new variables’ section.

## Results

### Descriptive statistics

Our final dataset of 2,133 data points combining survey and observational data was relatively balanced in gender, with 48% (1017 out of 2133) female and 52% (1116 out of 2133) male students. The age breakdown of the 2133 students in the dataset was as follows: 34% were 5 to 9 years old (730 students), 24% were 10–11 years old (515 students), 29% were 12–14 years old (609 students), and 13% were 15 years or older (279 students). For a comprehensive overview of the variables and their descriptive statistics, please refer to S1 Table for numeric variables and S2 Table for binary variables.

For children younger than 12, the average dmft was 1.62(2.26), and the average DMFT was 0.85(1.50). For adolescents 12 years old or older, average DMFT was 3.02(3.34). The average dmft/DMFT index was 2.17(2.62). Notably, the dmft/DMFT index fluctuated with age, reaching a low point around ages 10–12 when permanent teeth emerge and then increasing with age (see Fig 1).

**Fig 2:**
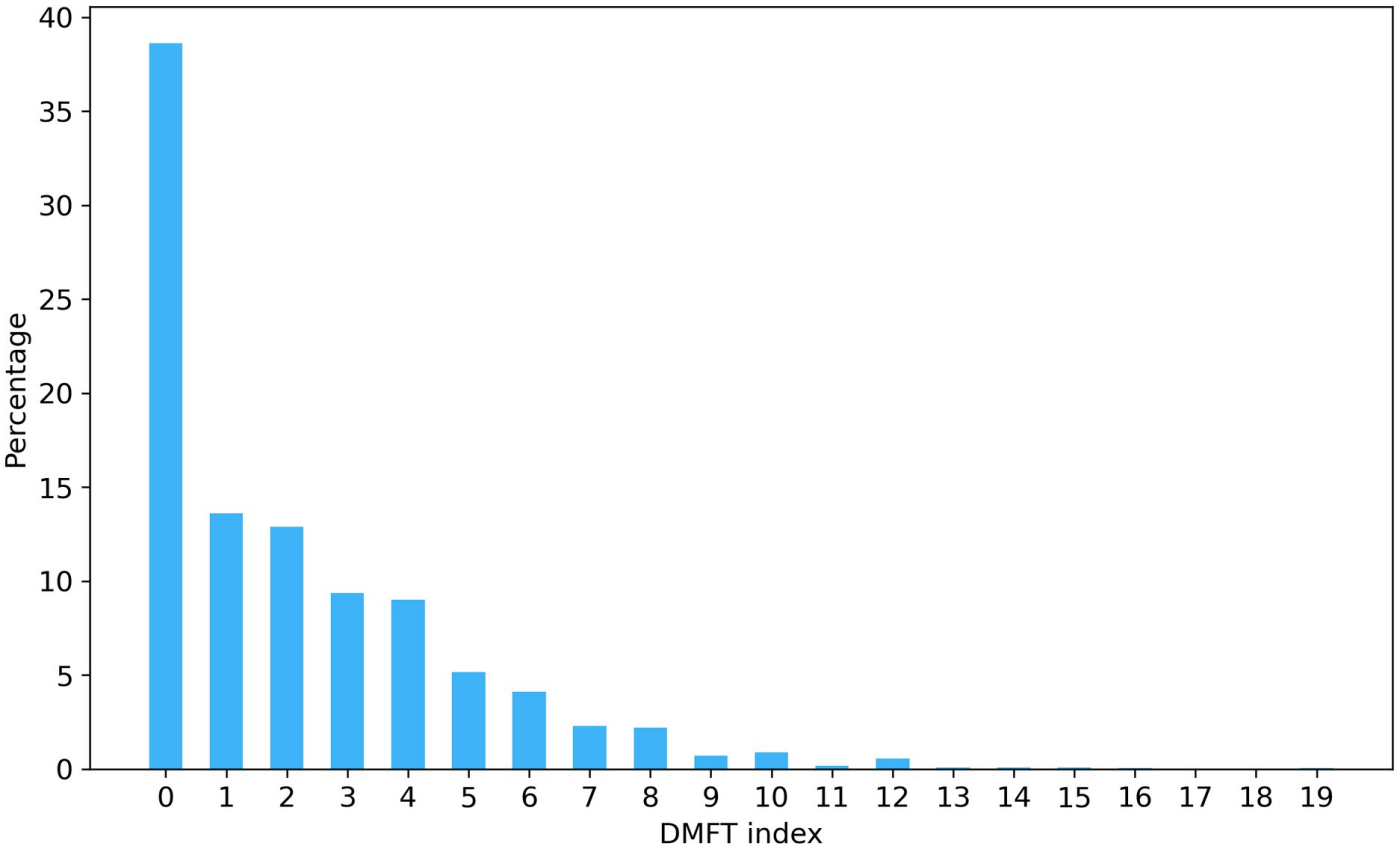
Distribution of students by dmft/DMFT index.

The analysis of the dmft/DMFT index distribution, presented in Fig 2, revealed that out of 2133 students, nearly 40% (824 students) of the sample had no decayed, missing, or filled teeth, 48% had at least two decayed, missing, or filled teeth (1019 students), and 26% had at least four decayed, missing, or filled teeth (544 students). Analysis of the data separately for children younger than 12 (for dmft and DMFT) and adolescents 12 years old or older (for DMFT) can be found in S2 File.

### Group comparisons using t-tests

The results of a series of independent-sample two-tailed t-tests suggested statistically significant and positive associations between certain oral health behaviors and better dmft/DMFT index values. We also compared these results with those obtained by conducting the tests separately in two subsamples, for children younger than 12 years (with dmft or DMFT) and for those 12 years old or older (DMFT only). Students who flossed had an average dmft/DMFT index value of 1.98(2.53), which was lower than the one for students who did not floss (2.46(2.70), *p* < 0.001). Testing these differences within the subsamples, we find that this difference is only present for students younger than 12 years for deciduous teeth (*p* = 0.003) (S3 File, Table 1.1) On average, students who reported frequent dental visits had a dmft/DMFT index value of 2.09 (2.53), which is lower than those who said they did not frequently visit the dentist (2.45(2.91), *p* = 0.009). Such a significant difference is, however, present only among students aged 12 or older (*p* = 0.002, S3 File, Table 3.1). There was no statistically significant difference in average dmft/DMFT index values between students who brushed their teeth at school (1.74(2.10)) and students who did not brush their teeth at school (2.20(2.65), *p* = 0.107). However, brushing at school was associated with lower DMFT (permanent teeth) for students younger than 12 years (*p* = 0.030, S3 File, Table 2.1). While 75% of the 2133 students in the sample reported regular dental visits (1602 students), 24% reported flossing (504 students), and 4% brushed their teeth at school (89 students).

Brushing teeth three times daily was associated with lower average dmft/DMFT index values of 1.88(2.52), compared with the average dmft/DMFT index of 2.23(2.67) (*p* = 0.019) for those brushing their teeth four or five times. We did not find a statistically significant difference in the average dmft/DMFT index value for students who brushed their teeth three times a day compared to students who brushed their teeth one or two times a day 2.20(2.65), *p* = 0.106. Considering the subsamples, a statistically significant group difference is noted only when comparing brushing three times with brushing once or twice daily (*p* = 0.009, S3 File, Table 1.1, and *p* = 0.041 S3 File, Table 3.1, for students younger than 12 and those 12 and older, respectively). Students who ate breakfast daily had an average dmft/DMFT index of 2.01(2.51), which is lower than the average dmft/DMFT index for students who skipped breakfast (2.79(2.92), *p* < 0.001). A similar and significant difference was observed only for those younger than 12 but for permanent teeth (*p* < 0.001, S3 File, Table 2.1) and for those aged 12 or older (*p* < 0.001, S3 File, Table 3.1). Students who consumed four to six meals per day had an average dmft/DMFT index of 2.07(2.52), which was lower than the average dmft/DMFT index value for students who ate fewer than four meals (2.62(2.98), *p* < 0.001). The analysis of subsamples provided support for this finding only among students aged 12 or older (*p* = 0.030, S3 File, Table 3.1). No statistically significant differences were found in the average dmft/DMFT index values for students who consumed four to six meals per day and those who ate more than six meals daily, who had an average dmft/DMFT score of 2.48(3.02), *p* = 0.131.

Students who frequently engaged in physical activity outside of school had an average dmft/DMFT Index value of 2.02(2.52), a value lower than those who rarely or never did so, who had an average dmft/DMFT index value of 2.43(2.74), *p* < 0.001. The same association holds only among those younger than 12 and for deciduous teeth (*p* < 0.001, S3 File, Table 1.1).

No statistically significant differences were found in the average dmft/DMFT index value for male 2.08(2.50) and female students 2.26(2.72), *p* = 0.124. However, we find a significant difference among students aged 12 or older, with females exhibiting higher DMFT values than males (p = 0.005, S3 File, Table 3.1). Students not receiving social assistance had an average dmft/DMFT value of 1.96(2.48), which was lower than the dmft/DMFT index for those without assistance, 2.62(2.82), *p* < 0.001, and 2.63(2.85), *p* < 0.001, for the highest and medium assistance levels, respectively. Similar and statistically significant differences are found for those younger than 12 for dmft (S3 File, Table 1.1), and for those aged 12 and older (DMFT, S3 File, Table 3.1).

Additional independent-sample two-tailed t-tests were conducted to compare the average consumption of distinct food items (processed sugary, dairy, fast-food, and healthy food) among students with dmft/DMFT values above and below the predefined thresholds (DMFT ≥ 3 or DMFT ≥ 4). Table 1 presents the averages and standard deviations of the consumption of each food group for the students with dmft/DMFT above or below the thresholds. Students with higher average dmft/DMFT scores consistently consumed a wider variety of sugary items than those with lower scores (*p* < 0.001 for both thresholds). Separate analysis shows similar and statistically significant results when considering only those younger than 12 and dmft and both thresholds (S3 File, Table 1.2), as well as those aged 12 or older and DMFT ≥ 3, but no significance was found for that group and DMFT ≥ 4 (S3 File, Table 3.2).

**Table 1:** Average distinct number of food items typically consumed in a day, per food category, for students with DMFT equal or above and those below pre-defined thresholds (DMFT ≥ 3 or DMFT ≥ 4).

DMFT above threshold		Sugary	Dairy	Salty fast-food	Healthy
DMFT ≥ 3	Yes	6.35(6.31)	5.55(3.12)	2.52(3.04)	7.41(3.82)
	No	5.20(4.90)	5.27(2.74)	2.10(2.65)	7.12(3.60)
DMFT ≥ 4	Yes	6.30(6.50)	5.49(3.16)	2.54(3.01)	7.35(3.87)
	No	5.33(5.02)	5.32(2.78)	2.15(2.72)	7.17(3.92)

Standard deviation is indicated in parentheses.

Additionally, students with dmft/DMFT ≥ 3 ate more distinct dairy and salty fast-food items (*p* = 0.035 and *p* < 0.001, respectively) than those with dmft/DMFT lower than 3. A similar difference was observed for salty fast-food consumption in students with dmft/DMFT ≥ 4 (*p* = 0.005). However, no differences were observed in dairy consumption (*p* = 0.229) between those with dmft/DMFT index below 4 and those with dmft/DMFT ≥4. Considering the group analysis within the subsamples, we observed statistically significant differences in the consumption of salty fast-food only for older children and both threshold levels (S3 File, Table 3.2) and not for younger children (S3 File, Table 1.2). We also see discrepancies when considering the consumption of dairy products. Like for the entire sample, the statistical significance was observed for younger students and a dmft threshold level of 3, but also a threshold level of 4 (S3 File, Table 1.2). However, no difference was observed for older students for both threshold levels (S3 File, Table 3.2). Also, no statistically significant differences were found in the number of distinct healthy items eaten by students with lower and those with equal or higher dmft/DMFT index when considering the dmft/DMFT = 3 (*p* = 0.081) and the dmft/DMFT = 4 thresholds (*p* = 0.313). However, when considering only the subsample of students younger than 12, we found statistically significant differences in the consumption of healthy food between students with higher and lower dmft scores (*p* < 0.001 for dmft ≥ 3 and p = 0.004 for dmft ≥ 4, S3 File, Table 1.2). No statistical significance was observed within the sample of students aged 12 and older.

### Model performance

Recall that our target variables were constructed using two dmft/DMFT index thresholds to identify students at risk of poor oral health: dmft/DMFT ≥ 3 (35%, 744 out of 2133 students) and dmft/DMFT ≥ 4 (25.5%, 544 out of 2133 students). We built a classification model for each of these variables, which we refer to as DMFT3 and DMFT4 models, respectively.

Among all trained and tested models, the best performing were two logistic regression models built with variables selected through low-variance filtering and recursive variables elimination [43]. For detailed model characteristics, please refer to S4 Table. Since our objective was the identification of individuals rather than explaining contributing factors, we omitted p-values in the subsequent analysis. Recursive elimination of variables, as detailed in the Methods section, was our primary tool for assessing the importance of independent variables. For complete lists of variables initially considered in the modeling process, see S1 Table (for numeric variables) and S2 Table (for binary variables).

Table 2 outlines the variables and their coefficients in the final DMFT3 and DMFT4 models. A negative coefficient indicates that an increase in that variable decreased the likelihood of a student exceeding the DMFT threshold. Variables not listed in the table were excluded from the final models. Notably, the DMFT4 model generally included coefficients with higher absolute values and fewer variables overall.

**Table 2:** Variables included in at least one regression model, after variables selection, and their respective coefficients.

Variable	Variable description	DMFT3 model coefficient	DMFT4 model coefficient
Age	Age of the student, measured in years	0.08	0.07
Age5to9	Whether the student is 5 to 9 years old	0.19	0.28
Age12to14	Whether the student is 12 to 14 years old	-	0.02
Age15+	Whether the student was older than 15 years	0.16	0.22
SocialAssistanceA	Whether the student receives the highest social assistance level	0.09	-
Breakfast	Whether the student usually has breakfast	0.09	0.15
SugarBreakfast	Count of processed sugar items consumed during breakfast	0.13	0.17
WaterLunch	Count of glasses of water typically drank at lunch	0.16	0.23
DairyAfternoonSnack	Count of dairy items consumed in the afternoon	-0.08	-0.17
DairyAfterDinner	Count of dairy items consumed after dinner	0.12	0.11
HowFrequentlyDentist	Frequency with which the student goes to the dentist	0.09	0.10
OftenBrushesWeekly	How often the student brushes their teeth in a typical week	-0.10	-0.11
Howlongbrushes3min	If the student says he/she brushes teeth for about 3 minutes	0.02	-
Howlongbrushes5min	If the student says he/she brushes teeth for about 5 minutes	0.03	-
HowlongbrushesDK	If the student does not know how long he/she brushes teeth for	0.07	-
HeardTartar	If the student has heard of an oral health disease called tartar	-0.08	-
KnowsFloss	If the student knows how to use dental floss	-0.09	-0.10

**Fig 3:**
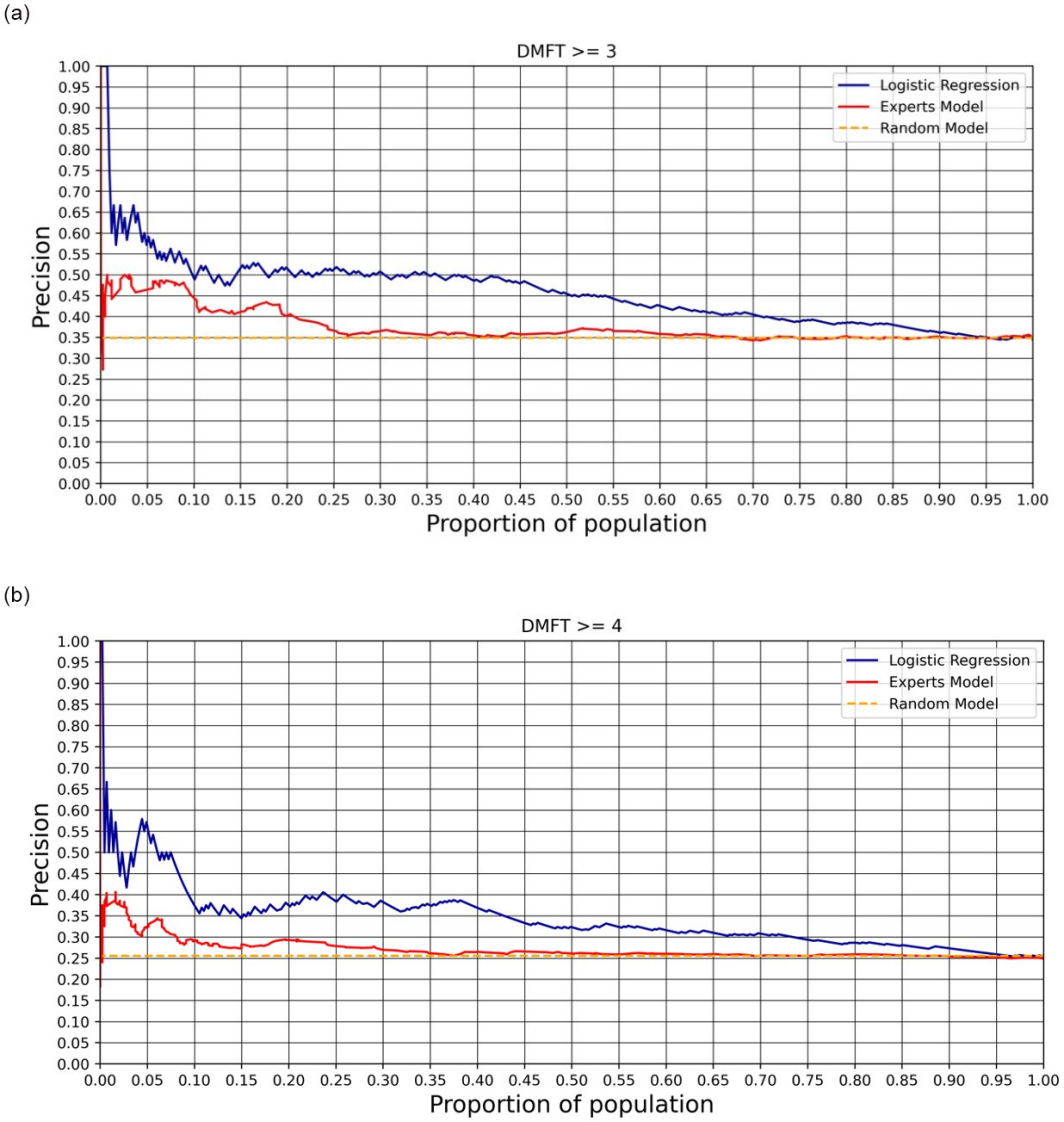
Precision@k for the (a) model estimating DMFT≥ 3 and (b) DMFT ≥ 4.

Fig 3 shows the comparison of our proposed model performance against two baselines: (a) a random model (simulating random student selection for intervention) and (b) a rule-based expert model (with rules defined by oral health professionals; for more details, see S1 File). The figure demonstrates the performance of each model at various levels of k.

An independent samples two-tailed t-test revealed that the average precision of the DMFT3 model 0.458(0.087), calculated over the entire range of k (0–100), was higher than the average precision of the experts’ model, 0.392(0.073), *p* < 0.001). The same result was verified for DMFT4; while the proposed model had an average precision of 0.347(0.082), the experts’ model had an average precision of 0.303(0.077), *p* < 0.001. As for the area under the curve (AUC), the DMFT3 model achieved an AUC of 0.64 and the DMFT4 model achieved an AUC of 0.65. For the corresponding receiver operating characteristic (ROC) curves, please refer to S1 Fig.

If we chose to intervene on only 10% of the student population (213 students), our DMFT3 model would identify students at high risk of poor oral health 1.4 times more effectively than random selection. It would also outperform an expert-based selection process by a factor of 1.1. The DMFT4 model had slightly better results, performing 1.4 times better than random selection and 1.2 times better than the expert-driven model. The DMFT3 and DMFT4 models showed high agreement, Cohen’s Kappa = 0.79. For more details on the two models, please refer to S4 Table.

#### Model performance per age group

Table 3 presents the mean precision of the proposed models, averaged over the entire range of k (0–100), and compares it to the mean precision of both the experts’ model and a random model, for the age groups and target variables considered in this study.

**Table 3:** Mean and standard deviation of the precision of the proposed models, calculated over the entire range of k (0–100) across different subsamples and considering dmft or DMFT as target variables.

Sample	Target variable	Model	Mean(standard deviation) of precision@k
Full sample	binary dmft/DMFT3	DMFT3 model	0.458(0.087)
Full sample	binary dmft/DMFT3	Experts’ model	0.392(0.073)
Full sample	binary dmft/DMFT3	Random model	0.345(0.000)
Full sample	binary dmft/DMFT4	DMFT4 model	0.347(0.082)
Full sample	binary dmft/DMFT4	Experts’ model	0.303(0.077)
Full sample	binary dmft/DMFT4	Random model	0.255(0.000)
Younger than 12 years	binary dmft3	DMFT3 model	0.384(0.110)
Younger than 12 years	binary dmft3	Experts’ model	0.203(0.863)
Younger than 12 years	binary dmft3	Random model	0.261(0.000)
Younger than 12 years	binary dmft4	DMFt4 model	0.281(0.097)
Younger than 12 years	binary dmft4	Experts’ model	0.138(0.084)
Younger than 12 years	binary dmft4	Random model	0.183(0.000)
Younger than 12 years	binary DMFT3	DMFT3 model	0.078(0.067)
Younger than 12 years	binary DMFT3	Experts’ model	0.110(0.078)
Younger than 12 years	binary DMFT3	Random model	0.097(0.000)
Younger than 12 years	binary DMFT4	DMFT4 model	0.047(0.065)
Younger than 12 years	binary DMFT4	Experts’ model	0.083(0.078)
Younger than 12 years	binary DMFT4	Random model	0.062(0.000)
12 years or older	binary DMFT3	DMFT3 model	0.494(0.090)
12 years or older	binary DMFT3	Experts’ model	0.444(0.082)
12 years or older	binary DMFT3	Random model	0.388(0.000)
12 years or older	binary DMFT4	DMFT4 model	0.401(0.092)
12 years or older	binary DMFT4	Experts’ model	0.351(0.093)
12 years or older	binary DMFT4	Random model	0.306(0.000)

As shown in Table 3, the machine-learning models proposed in the current study outperformed the experts’ model when considering children younger than 12 years and dmft as the target variable. When considering the dmft ≥ 3 threshold, the average precision of the experts’ model, calculated over all values of k (0–100) was 0.202(0.863) and the average precision of the proposed model was 0.384(0.110). When considering the dmft ≥ 4 threshold, the average precision of the experts’ model was 0.139(0.084) and the average precision of the proposed model was 0.281(0.097). A t-test confirmed the differences between our model(s) against the benchmarks were statistically significant (*p* < 0.001 for both comparisons). Similar results were found for adolescents 12 or older when considering DMFT as the target variable. The average precision of the experts’ model was 0.444(0.082) and 0.351(0.093) for the DMFT3 and DMFT4 thresholds, respectively, while the performance of the proposed models was 0.494(0.090) and 0.401(0.092), respectively. Both differences weres statistically significant (*p* < 0.001). However, results show that our models performance was worse than random for children younger than 12 when considering the DMFT index as the target variable, as well as worse than the experts’ model (*p* < 0.001 for all comparisons).

Analyses of the AUC also suggest that the application of the proposed models to the subsamples of children younger than 12 years (considering binary dmft) and adolescents aged 12 or older (considering binary DMFT) did not diminished the performance of the models. While the AUC of the DMFT3 model when applied to the entire sample was 0.64, it reached 0.69 when the model was applied to children younger than 12 years and was the same (0.64) for adolescents aged 12 or older. The AUC of the DMFT4 model was 0.65 when applied to the entire sample, 0.70 when applied to children younger than 12 years and 0.63 when applied to adolescents aged 12 or older. We note, however, that the AUC of the models when applied to children younger than 12 and considering the binary DMFT was below 0.50.

We present the corresponding precision curves and the ROC curves plots separately for each age group in S4 and S5 Files, respectively.

### Bias analysis

**Fig 4:**
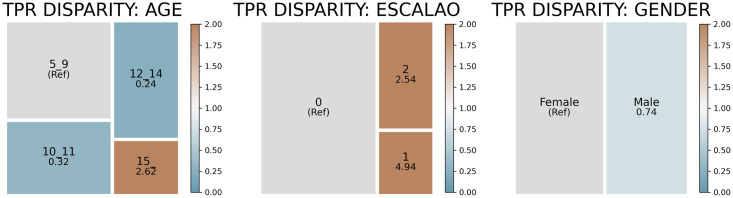
TPR disparity of the DMFT3 model when the 10% of the population with the highest model scores is selected as being at risk of poor oral health.

**Fig 5:**
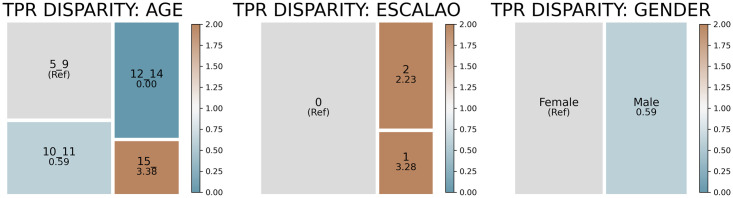
TPR disparity of the DMFT4 model when the 10% of the population with the highest model scores is selected as being at risk of poor oral health.

Figs 4 and 5 present the results of the bias analysis for the DMFT3 and DMFT4 models. Box sizes indicate the number of students within each group. The color intensity indicates the intensity of disparity relative to the majority group (shown in grey). Brown boxes show groups with higher TPRs (more likely to receive intervention), while blue boxes indicate lower TPRs (potentially overlooked groups).

Bias auditing revealed potential biases within the models. If we select the top 10% of students with the highest estimated scores of having a dmft/DMFT index higher than the threshold, both models are less likely to identify correctly male students than female students. Additionally, students receiving social assistance, an indicator of economic vulnerability, were 2 to 5 times more likely to be selected than students with a dmft/DMFT index higher than the threshold who did not receive social assistance. We also observed an age bias: those over 15 or under 10 with poor oral health were more likely to be correctly selected than those in intermediate age groups.

**Fig 6:**
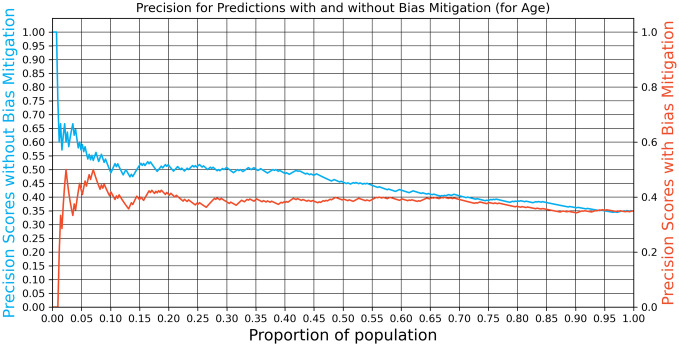
Precision@k with and without bias mitigation (DMFT3 model).

**Fig 7:**
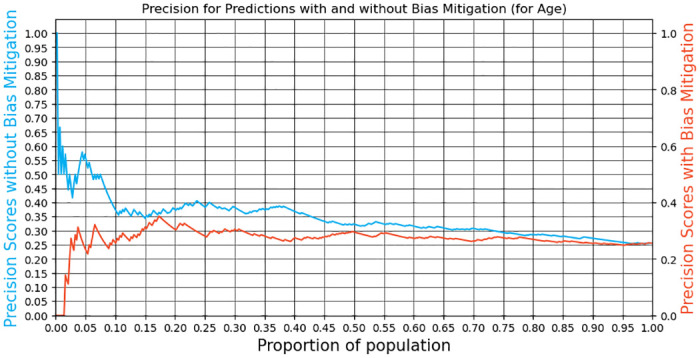
Precision@k with and without bias mitigation (DMFT4 model).

Given these disparities, we explored bias mitigation using the recall equalization technique [44]. While this technique did equalize true positive rates (TPR) across groups, it significantly reduced model performance. For the DMFT3 model, we saw a 10 and 15 percentage point drop in performance at the 5% and 10% high-risk thresholds (see Fig 6). The DMFT4 model exhibited an even more significant drop of 10 and 30 percentage points for the two thresholds, respectively (see Fig 7).

We conducted bias audit of the two models also by executing it on two subsamples, one with children younger than 12 (using dmft as the dependent variable) and the other with adolescents aged 12 or older (using DMFT as the dependent variable), In both cases we compared the TPR between groups based on age, gender and social assistance. We did not perform bias analysis for students younger than 12 using DMFT as the dependent (target) variable as the model performed very poorly, as shown in the previous section. As elaborated in S6 File, the audit results revealed similar patterns of bias when the models were applied to the subsamples, with more pronounced disparities observed among the groups. Furthermore, females tended to have a higher TPR than males when the models were applied to adolescents aged 12 or older.

## Discussion

The results suggest that we can reject the null hypothesis. There was a statistically significant improvement in the precision of identifying students with poor oral health (or having a DMFT index higher than the pre-defined threshold) when using machine learning models that use only self-reported survey data, compared to a rule-based model constructed with healthcare professionals. This superior performance of the machine learning models, compared with rule-based experts’ models, was evident when considering the entire sample of students (average precision 0.458 vs. 0.392 for the proposed DMFT3 and experts’ model, respectively, and 0.347 and 0.303 for the proposed DMFT4 and experts’ model, respectively), as well as in the separate analysis of the models in sub-samples. For students younger than 12 years (considering binary dmft indices as the target variables), average precision was 0.384 vs 0.202 for the DMFT3 and the experts’ model, respectively, and 0.281 vs 0.139 for the DMFT4 threshold. For the subsample of students 12 years old or older, considering binary DMFT indices as the target variable, average precision was 0.494 vs 0.444 for the DMFT3 and the experts’ model, respectively, and 0.401 and 0.351 for the DMFT4 threshold. Importantly, no significant deterioration of performance of the models was verified when applying the proposed models to the children younger than 12 (considering the binary dmft indices) and adolescents aged 12 or older (considering the binary DMFT indices) subsamples, as measured by the AUC, which ranged from 0.63 to 0.70.

These results add to the body of evidence of the usability of systems for conducting oral health prescreening using only self-reported data [6, 9, 11]. This study, given its unique context of students from 5 to 19 years old, signals the potential of self-reported behavioral data to estimate the oral health of children and adolescents and the usability of such an approach in proactive screening programs. This proactive approach is an excellent policy tool for oral health monitoring and interventions planing when the aim is to improve oral health outcomes for students affordably and within the realities of the dental care system.

Before we elaborate on a potential use case, let us reflect upon the results in light of the existing work. The model(s) proposed in this study demonstrated slightly lower precision than the other models we found in the literature [7, 9, 20]. The performance of our selected models is below the lower end of the AUC range observed in the literature for different caries risk assessment tools (0.74–0.97) [6, 45, 46]. There are two likely explanations for such a performance result. The first explanation is related to the specification of the target variable. In some cases, the “high risk” is defined as having DMFT higher than one [6, 9], while in our case, the threshold is set to equal or higher than three or four. This translates to fewer observations of individuals with a value of 1 in the target (dependent) variable and, therefore, a more challenging modeling task to achieve high precision. The second explanation is that the target population in this study includes both children and adolescents and is not directly comparable with the other studies that propose similar approaches for specific population groups, such as preschool children [6] or only 12 years old adolescents [9]. Despite these challenges, the results suggest substantially better performance than the model constructed in collaboration with the panel of experts or the random choice model and are comparable to the AUC range (0.62–0.64) obtained by other survey-based models applied to children and adolescents [22].

Separate analysis for children younger than 12 years suggest that the models were not suitable to adequately predict their DMFT index. This is likely due to the transitional phase between deciduous and permanent dentition in this age group, as time is required for caries to develop in recently erupted permanent teeth. Mean DMFT index value in this subsample is lower (0.85) than their mean dmft index values (1.62). As the dmft index in younger years tends to be an important predictor of the DMFT index in early-adolescence [24], using only the dmft index serves as a good measure for prioritizing children for proactive oral health interventions.

The observed mean dmft/DMFT index (for the entire sample) was 2.17, while the average DMFT observed among adolescents (12 years only) was 3.02. The adolescents’ average DMFT value is significantly higher than the average values observed in Brazil (2.1) in 2010 [9] and globally, as reported by the World Health Organization in 2000 (2.3) [47]. While slightly biased, the advantage of working with such a sample is that there are more cases of poor oral health, and therefore, the trained models are more likely to capture patterns in the data that drive such an outcome. It is also possible that these drivers are idiosyncratic to the sample, which calls for further investigations of the generalizability of the approach at national and international levels.

Considering the important variables that were used for the identification of students with high dmft/DMFT index, which we established by the variables selection process, we observed several similarities to the existing studies. The models’ ability to identify the oral health status of children based solely on demographic and behavioral data is aligned with the existing literature showing that socioeconomic conditions, demographic characteristics, and oral health behaviors are correlated with oral health not only of adults [11, 48, 49] but also, and perhaps especially, of children [9, 10, 22, 50, 51]. Karhade et al., [6] show that a parsimonious model comprised of only two factors outperforms more complex models in identifying students with current caries; these two factors were children’s age and their parents-reported child oral health status. In our study, age has also emerged as an important factor. Bomfim [9] emphasizes the use of dental floss and unhealthy food consumption as important variables for identifying 12-year-old individuals with caries, the categories of variables that we have also found to be important for identifying school children with a high dmft/DMFT index. The same author did not specify the importance of age and reported that the model trained by the XGBoost machine learning algorithm outperforms logistic regression. Other studies of a similar character, although not with the same target (dependent) variable, also reported complex algorithms outperforming logistic regression [10, 21, 22]. In our case, however, the results are more aligned with the findings of Christodoulou et al., [52], who studied 71 journal reports and found no substantial performance benefits of alternative machine learning models for caries prediction/identification to logistic regression. We found the logistic regression to outperform all other alternative machine learning models. Particularly notable is that the variables used in the logistic regression models include the awareness of oral health issues (i.e., tartar) or the knowledge about techniques for teeth maintenance, such as cognition about flossing. While also being present in our model, cognition about flossing was considered as part of the mode for predicting active caries and dentist referral [22].

We compared the approach proposed in this study to a selection of six other studies employing machine learning for caries risk and current status assessment, considering the groups of variables used, their respective target populations, and performance metrics. The comparison results are provided in Table 4. As discussed above, the proposed model can offer comparable results even when requiring less and easier-to-obtain information. The most important strength of the proposed model is that it depends solely on self-reported data and a few groups of variables that could be equally applied to children and adolescents. In contrast to the trends of developing complex models and increasing the extensiveness of data, this study echoes the calls for a return to practical, streamlined approaches that can enhance healthcare system performance within the constraints of limited resources and data availability [9–11].

**Table 4:** Comparison of the variables included in models for caries risk and current status estimation.

Variables	Current model	[7]	[22]	[19]	[53, 54]	[55]
**Sociodemographic variables**						
Demographic variables	x		x	x		
Socioeconomic status	x	x	x	x		
**Survey variables**						
Oral health habits and knowledge	x	x	x			
Oral health symptoms		x	x	x		
Diet	x				x	x
Current caries experience		x	x		x	
**Clinical variables**						
Current caries experience					x	
Past caries experience				x	x	x
White spot lesions					x	
Pits and fissures					x	
Orthodontic appliances					x	
Systemic health					x	x
Medication					x	
Cariostat score						
Calculis				x		
Flurosis				x		
**Clinical images**				x		
**Salivary & Microbiological variables**						
Plaque variables					x	x
Saliva flow rate					x	
Saliva buffering capacity						
Mutans streptococci					x	
Lactobacilli					x	
Genetic variables						
**Population**	Children&Adolescents	Adolescents	Children&Adolescents	Children	Children&Adults	Children
**Performance**	AUC 0.64–0.65	AUC 0.86	AUC 0.62–0.64	AUC 0.90	Precision 0.64–0.76	AUC 0.72

Note: We exclude the models from [5, 20] from the above table as the authors do not clearly specify the variables used in the model.

Let us consider a use case for the proposed model utilizing a real-world example. In countries like Portugal, the standard practice involves students accessing oral care through checkups in primary care facilities, school-based screenings, or vouchers for private care when public primary care lacks oral health services. With limited oral care capacity in many public primary care facilities, but also given the inconclusive effects of in-person visits to dentists [13], vouchers have become the main access provision mechanism for oral care. However, voucher underutilization among the children and adolescent population in Portugal [12], along with infrequent checkups for everyone and absence from scheduled visits to dental centers in Brazil [56], suggests that there is a high need for alternative preventive strategies to improve oral health. Our study echoes previous calls [6, 7, 22, 46] for targeted, proactive, cost-effective oral health management approaches for students based on survey data.

Scaling a proactive approach by non-targeted messaging of the entire student population is appealing but faces practical constraints. Consider, for instance, using universal messaging to motivate students to book check-ups. Such an approach could overwhelm the system’s capacity if there are many scheduled check-ups. Conversely, relying solely on the initiative of schoolchildren or their guardians, as in Portugal’s voucher system, risks primarily reaching those already invested in their oral health. Therefore, there is a high potential to end up with sub-optimal results despite significant investment. A possible answer to that challenge is in need-based planning and resource allocation. That approach would include the screening of schoolchildren and a low-cost assessment (e.g., survey-based) of their current oral health, and upon the results verification (e.g., using triangulation, reliability, and validity testing), the development of strategies that would make a need-based distribution of oral care services.

Consider a hypothetical scenario where a policymaker decides to invest in targeted campaigns, and the healthcare units have estimated their capacity to receive 10% of the student population over the next six months for clinical examination. In that case, the machine learning model proposed in this study offers distinct societal benefits. If the surveys have been collected regularly, which is relatively cheap, identifying students likely to have poor oral health using ML models is easier and more effective than any existing alternative. By focusing on the available capacity (the 10% identified as having poor oral health) and over six months, limited dental resources would be used to provide access to services to those who are likely to need the treatment. Those in need could be contacted multiple times at the school or through guardians, and offered free checkups. Timely preventive interventions could reduce the need for complex, expensive future treatments, decreasing healthcare costs. Moreover, the model’s reliance on self-reported data promotes equity by making it adaptable to resource-limited settings where clinical assessments may be scarce; for example, fewer dentists are available in rural areas, and therefore, there tend to be fewer checkups.

Analysis of the group differences using t-tests offers insights into the potential poor oral health risk factors for different age groups and those likely to be shared among all students. These factors can be considered when planing targeted interventions among different risk groups as identified with the proposed approach.

The approach proposed in this article offers a possibility of addressing ethical concerns, which often get little explicit attention. In high-stakes scenarios like healthcare, where decisions can significantly impact students’ access to care and well-being, ethical considerations surrounding ML are paramount. Of particular concern are the interpretability and fairness of machine learning models. Interpretability refers to explaining how a model arrives at a specific result, allowing humans to oversee and assess the model’s outputs [57]. Fairness is usually operationalized to ensure that models do not introduce or perpetuate biases that may further disadvantage certain groups within the population [44].

The model evaluation yielded the logistic regression model as the best-performing one. Logistic regression models are inherently interpretable, so the “black box” issue often associated with more complex ML algorithms is irrelevant. Each decision is transparent and relatively easy to understand. This aligns with ethical considerations in healthcare and proposed regulations like the EU Artificial Intelligence Act, which emphasizes the importance of interpretability in such applications.

Even with interpretable models, any system designed to improve oral health can potentially exacerbate existing inequalities. It is essential to proactively audit models for biases related to factors like economic status, age, or gender [58–60]. Although bias auditing is uncommon in oral health literature, such practice is required to inform stakeholders and policymakers, enabling them to make well-reasoned decisions about model deployment with full awareness of potential biases [44]. This study calls for more evidence of the bias of different caries risk assessment or screening tools and their underlying approaches.

Our bias auditing revealed that both models favor specific student groups: those receiving social assistance, younger students (5–9 years), and older students (15+ years). When applied to the sub-sample of adolescents aged 12 or older, the models also tended to favor female students. While adjustments could potentially address these biases, our analysis indicates this correction would significantly diminish model precision by 5 to 15 percentage points (for interventions targeting more than 10% of the student population). This trade-off contrasts with findings in other high-stakes settings [44], highlighting the need for careful consideration by stakeholders and decision-makers—a challenge worthy of a separate study.

One limitation of this study is the relatively small dataset compared to big data projects [8]. Scaling the proposed model to other school districts would naturally increase the data volume and likely enhance precision by uncovering less common patterns within the data.

Further research on the use of survey data and machine learning for caries risk assessment or screening could go in two directions. One direction is to go towards parsimony, and like in the work of Karhade et al. [6], explore the minimal number of variables in a model that would not penalize the model’s performance. The other direction is to go towards better quality of the identification and explore incorporating additional variables that potentially impact oral health. Expanding the survey by increasing the number of questions and the explored dimensions may lead to better model performance. Additionally, tailoring surveys to specific age groups could reduce misunderstanding or the mixture of different dimensions (dmft/DMFT) and improve data quality. It is important to acknowledge the potential for bias in self-reported data and the possibility of concept drift. Also, our model was trained on self-reported data at specific time points. Periodic monitoring and retraining may be needed as external factors influence the underlying population and oral health outcomes.

This study relied on a single survey instance, limiting our model to identifying students with a DMFT exceeding a given threshold rather than its potential increase over time. Longitudinal studies with multiple screenings per student would enable such predictions, allowing for targeted prevention among those at risk of oral health deterioration. This would increase the model’s clinical relevance and performance. Additionally, while the data were collected from all schools in a municipality, that is still a single region with similar distal factors. Hence, there is a need for more studies with international coverage and more diversity.

While collecting additional data, tailoring surveys, and incorporating a longitudinal component could improve model precision, they also introduce complexity and resource demands. Future research could compare the current system (oral screenings by professionals for some students) with the proposed ML-based system (using self-reported data and scalable to all students) through, for instance, a pilot study and a cost-benefit analysis. This comparison should assess accuracy, false negatives, treatment access speed, adherence, potential cost savings, and health outcome improvements. By investigating these trade-offs, policymakers can make evidence-based decisions about the cost-effectiveness of different interventions.

## Conclusion

This study demonstrated the feasibility of developing a machine-learning model to identify children and adolescents with poor oral health using only survey data and relatively few variables, with performance comparable to the more complex models. It adds to the body of literature that studies the use of machine learning for caries risk assessment or identification, offering evidence of the applicability of this approach to assist a broad population of five to 19-year-old students. We argue that the proposed approach is important for policies to help avoid self-selection of those already concerned for their oral health and increase the reach of interventions. Additionally, bias audit of the trained model is a contribution on its own. Although bias auditing is becoming fairly standard in other fields, it is still not a common practice among oral health scholars, despite their growing interest in the use of machine learning and artificial intelligence. We have explored possible remedies to the observed bias. However, given the moderate performance of our model, bias remained a challenge. Therefore, this study also points to the necessity of assessing the trade-off between the intended effects related to the optimization of resources and better oral health outcomes and the unintended effects of unfair allocation of oral health care services, especially for the disadvantaged. In practical terms, policymakers should monitor for biases in outcomes and plan interventions to deal with them as implementations of policies unfolds. Regardless of whether the researchers decided to take the direction of parsimony (fewer variables) or completeness (more variables) of the models, as Halasa-Rappel et al., [61] suggest, there is a need to improve the validity of the proposed caries risk assessment and screening tools, decreasing the variability in their observed performance, and better understanding when and which models are more applicable [46]. This is an important pursuit for data-driven policies in oral health and clinical decision-making.

While there are increasingly many studies that demonstrate the feasibility and value of low-cost and affordable machine-learning-based screening of children and adolescents using only survey data to classify their caries status and optimize resource use, the actual implementation of such models and the results of their applications are yet to be observed. As Damschroder et al., [62] suggest, there is a need for joint work among scholars and policymakers globally to advance the implementation science and understand what works and what does not work. Implementation is a critical factor for advancing this branch of oral health management in general and in particular for children and adolescents.

Therefore, let us conclude this article with a call for scholars, practitioners, and policymakers to engage in purposeful experimentation and accumulation of shared evidence of the type of studies and questions, the performance of machine learning and AI models in practice, their intended and unintended benefits and potential harms, the interventions to manage potential disparities, and in that way contributing to the goals of the World Health Organization Global Oral Health Program, of building healthy communities and populations and combat ill-health [47].

## Supporting information

S1 Table Characterization of the numeric variables included in the project. (DOCX)

S2 Table Characterization of the binary variables included in the project. (DOCX)

S3 Table Food items classification. (DOCX)

S4 Table Characteristics of the best DMFT3 and DMFT4 models. (DOCX)

S1 Fig ROC curve plots for the DMFT3 and DMFT4 models, computed for the entire students’ sample. (DOCX)

S1 File Detailed description of the experts’ model. (DOCX)

S2 File Figures presenting the distribution of students by DMFT (separately for adolescents aged 12 or older and children younger than 12) and by dmft (for children younger than 12). (DOCX)

S3 File Tables presenting the association of DMFT (separately for adolescents aged 12 or older and children younger than 12) and dmft (for children younger than 12) with demographic, oral health, physical activity, and nutrition behavior variables. (DOCX)

S4 File Precision curves for the DMFT3 and DMFT4 models, computed separately for adolescents 12 or older (using binary DMFT as the dependent variable) and children younger than 12 (using binary dmft and binary DMFT as dependent variables). (DOCX)

S5 File ROC curve plots for the DMFT3 and DMFT4 models, computed separately for adolescents 12 or older (using binary DMFT as the dependent variable) and children younger than 12 (using binary dmft and binary DMFT as dependent variables). (DOCX)

S6 File Bias analysis plots for the DMFT3 and DMFT4 models, computed separately for adolescents 12 or older (using binary DMFT as the dependent variable) and children younger than 12 (using binary dmft and binary DMFT as dependent variables). (DOCX)

## References

[1] Kassebaum N, Bernabé E, Dahiya M, Bhandari B, Murray C, Marcenes W. Global burden of untreated caries: a systematic review and metaregression. Journal of Dental Research. 2015;94(5):650–8. doi: 10.1177/0022034515573272 25740856

[2] Kopycka-Kedzierawski D, Billings R. Prevalence of dental caries and dental care utilisation in preschool urban children enrolled in a comparative-effectiveness study. European Archives of Paediatric Dentistry. 2011;12:133–8. doi: 10.1007/BF03262794 21640057 PMC3111947

[3] Dettori M, Arghittu A, Cappai A, Castiglia P, Campus G, Group CSS. Impact of socioeconomic inequalities on dental caries status in Sardinian children. Children. 2024;11(1):96. doi: 10.3390/children11010096 38255409 PMC10814925

[4] Newacheck PW, Hughes DC, Hung YY, Wong S, Stoddard JJ. The unmet health needs of America’s children. Pediatrics. 2000 Apr;105(4 Pt 2):989–97. doi: 10.1542/peds.105.S3.989 10742361

[5] Pang L, Wang K, Tao Y, Zhi Q, Zhang J, Lin H. A new model for caries risk prediction in teenagers using a machine learning algorithm based on environmental and genetic factors. Frontiers in Genetics. 2021 Mar;12:636867. doi: 10.3389/fgene.2021.636867 33777105 PMC7990890

[6] Karhade D, Roach J, Shrestha P, et al. An automated machine learning classifier for early childhood caries. Pediatr Dent. 2021;43(3):191–7. 34172112 PMC8278225

[7] Levin L, Shpigel I, Peretz B. The use of a self-report questionnaire for dental health status assessment: a preliminary study. British Dental Journal. 2013 Mar;214(5):E15. doi: 10.1038/sj.bdj.2013.224 23470417

[8] Hung M, Voss MW, Rosales MN, Li W, Su W, Xu J, et al. Application of machine learning for diagnostic prediction of root caries. Gerodontology. 2019;36(4):395–404. doi: 10.1111/ger.12432 31274221 PMC6874707

[9] Bomfim RA. Machine learning to predict untreated dental caries in adolescents. BMC Oral Health. 2024 12;24:1–6. doi: 10.1186/s12903-024-04073-4 38461227 PMC10924973

[10] Qu X, Zhang C, Houser SH, Zhang J, Zou J, Zhang W, et al. Prediction model for early childhood caries risk based on behavioral determinants using a machine learning algorithm. Comput Methods Programs Biomed. 2022 12;227. doi: 10.1016/j.cmpb.2022.107221 36384058

[11] Çiftçi BT, Aşantoğrol F. Utilization of machine learning models in predicting caries risk groups and oral health-related risk factors in adults. BMC Oral Health. 2024 12;24:1–19. doi: 10.1186/s12903-024-04210-z 38589865 PMC11000438

[12] Simões J, Augusto GF, do Céu A, Ferreira MC, Jordão M, Calado R, et al. Ten years since the 2008 introduction of dental vouchers in the Portuguese NHS. Health Policy. 2018;122(8):803–7. doi: 10.1016/j.healthpol.2018.07.013 30054096

[13] Arora A, Nagraj SK, Khattri S, Ismail NM, Eachempati P. School dental screening programmes for oral health. The Cochrane database of systematic reviews. 2022 7;7. doi: 10.1002/14651858.CD012595.pub4 35894680 PMC9327802

[14] Featherstone J, Chaffee B. The evidence for caries management by risk assessment (CAMBRA^®^). Advances in Dental Research. 2018;29(1):9–14. doi: 10.1177/0022034517736500 29355423 PMC5784484

[15] Holgerson PL, Twetman S, Stecksèn-Blicks C. Validation of an age-modified caries risk assessment program (Cariogram) in preschool children. Acta Odontologica Scandinavica. 2009;67(2):106–12. doi: 10.1080/00016350802714734 19152150

[16] Hänsel Petersson G, Twetman S, Bratthall D. Evaluation of a computer program for caries risk assessment in schoolchildren. Caries Research. 2002 10;36(5):327–40. doi: 10.1159/000065963 12399693

[17] Cagetti MG, Bontà G, Cocco F, Lingstrom P, Strohmenger L, Campus G. Are standardized caries risk assessment models effective in assessing actual caries status and future caries increment? A systematic review. BMC Oral Health. 2018 Jul;18(1). doi: 10.1186/s12903-018-0585-4 30012136 PMC6048716

[18] Senneby A, Mejàre I, Sahlin NE, Svensäter G, Rohlin M. Diagnostic accuracy of different caries risk assessment methods. A systematic review. Journal of Dentistry. 2015;43(12):1385–93. doi: 10.1016/j.jdent.2015.10.011 26493112

[19] Ngnamsie Njimbouom S, Lee K, Kim JD. MMDCP: Multi-modal dental caries prediction for decision support system using deep learning. International Journal of Environmental Research and Public Health. 2022;19(17). doi: 10.3390/ijerph191710928 36078635 PMC9518085

[20] Kang IA, Ngnamsie Njimbouom S, Lee KO, Kim JD. DCP: prediction of dental caries using machine learning in personalized medicine. Applied Sciences. 2022;12(6):3043. doi: 10.3390/app12063043

[21] Wang Y, Hays RD, Marcus M, Maida CA, Shen J, Xiong D, et al. Developing children’s oral health assessment toolkits using machine learning algorithm. 2019 11;5:233–43. doi: 10.1177/2380084419885612 PMC7298887 31710817

[22] Xiong D, Marcus M, Maida CA, Lyu Y, Hays RD, Wang Y, et al. Development of short forms for screening children’s dental caries and urgent treatment needs using item response theory and machine learning methods. PLOS ONE. 2024 3;19:e0299947. doi: 10.1371/journal.pone.0299947 38517846 PMC10959356

[23] Costacurta M, Epis M, Docimo R, et al. Evaluation of DMFT in paediatric patients with social vulnerability conditions. European Journal of Paediatric Dentistry. 2020;21(1):70–3. 32183533 10.23804/ejpd.2020.21.01.14

[24] Motohashi M, Yamada H, Genkai F, Kato H, Imai T, Sato S, et al. Employing dmft score as a risk predictor for caries development in the permanent teeth in Japanese primary school girls. Journal of oral science. 2006;48(4):233–7. doi: 10.2334/josnusd.48.233 17220622

[25] Nora AD, da Silva Rodrigues C, de Oliveira Rocha R, Soares FZM, Minatel Braga M, Lenzi TL. Is caries associated with negative impact on oral health-related quality of life of pre-school children? A systematic review and meta-analysis. Pediatric dentistry. 2018;40(7):403–11. 31840639

[26] Shulman JD, Cappelli DP. Chapter 1—Epidemiology of dental caries. In: Cappelli DP, Mobley CC, editors. Prevention in Clinical Oral Health Care. Saint Louis: Mosby; 2008. Available from: https://www.sciencedirect.com/science/article/pii/B9780323036955500057.

[27] Robinson PG. Dental epidemiology. In: Quah SR, editor. International Encyclopedia of Public Health. Second edition. Oxford: Academic Press; 2017. p. 258–63. Available from: https://www.sciencedirect.com/science/article/pii/B9780128036785001041.

[28] Silva R, Assaf A, Mialhe F, Cortellazzi K, Meneghim M, Pereira AC. Dental caries diagnostic thresholds: Which one? Why? When? International Journal of Public Health. 2020;65. doi: 10.1007/s00038-020-01332-3 32016489

[29] World Health Organization. Oral health surveys: basic methods. 5th ed. WHO Press; 2013.

[30] McKinney W. Data structures for statistical computing in python. In: S van der Walt, J Millman, editors. Proceedings of the 9th Python in Science Conference; 2010. p. 56 61.

[31] Hunter JD. Matplotlib: A 2D graphics environment. Computing in Science & Engineering. 2007;9(3):90–5. doi: 10.1109/MCSE.2007.55

[32] Virtanen P, Gommers R, Oliphant TE, Haberland M, Reddy T, Cournapeau D, et al. SciPy 1.0: fundamental algorithms for scientific computing in python. Nature Methods. 2020;17:261–72. doi: 10.1038/s41592-019-0686-2 32015543 PMC7056644

[33] Pedregosa F, Varoquaux G, Gramfort A, Michel V, Thirion B, Grisel O, et al. Scikit-learn: machine learning in Python. J Mach Learn Res. 2011 nov;12(null):2825–2830. doi: 10.5555/1953048.2078195

[34] Cagetti MG, Bontà G, Lara JS, Campus G. Caries risk assessment using different Cariogram models. A comparative study about concordance in different populations—Adults and children. PLOS ONE. 2022;17(6):1–11. doi: 10.1371/journal.pone.0264945 35749436 PMC9231745

[35] Foster I, Ghani R, Jarmin R, Kreuter F, Lane J. Big Data and Social Science: Data Science Methods and Tools for Research and Practice; 2020. doi: 10.1201/9780429324383

[36] Chouldechova A. Fair prediction with disparate impact: a study of bias in recidivism prediction instruments. Big Data. 2017;5(2):153–63. doi: 10.1089/big.2016.0047 28632438

[37] Mehrabi N, Morstatter F, Saxena N, Lerman K, Galstyan A. A survey on bias and fairness in machine learning. ACM Computing Surveys (CSUR). 2021;54(6):1–35. doi: 10.1145/3457607

[38] Saleiro P, Kuester B, Hinkson L, London J, Stevens A, Anisfeld A, et al. Aequitas: a bias and fairness audit toolkit. arXiv preprint arXiv:181105577. 2018. 10.48550/arXiv.1811.05577

[39] Lamba H, Rodolfa KT, Ghani R. An empirical comparison of bias reduction methods on real-world problems in high-stakes policy settings. ACM SIGKDD Explorations Newsletter. 2021;23(1):69–85. doi: 10.1145/3468507.3468518

[40] Banu A, Șerban C, Pricop M, Urechescu H, Vlaicu B. Dental health between self-perception, clinical evaluation and body image dissatisfaction–a cross-sectional study in mixed dentition pre-pubertal children. BMC Oral Health. 2018;18:1–9. doi: 10.1186/s12903-018-0542-2 29724206 PMC5934803

[41] Gören BD, Derelioğlu SŞ, Yılmaz S. Assessing the Clinical Consequences of Untreated Caries in 8-to 10-Year-Old Children with Pufa Index. Journal of Advanced Oral Research. 2022;13(1):105–12. doi: 10.1177/23202068221075964

[42] Calado R, Ferreira C, Nogueira P, Melo P. Caries prevalence and treatment needs in young people in Portugal: the third national study. Community Dent Health. 2017;34(2):107–11. 28573842 10.1922/CDH_4016Calado05

[43] Guyon I, Weston J, Barnhill S, Vapnik V. Gene selection for cancer classification using support vector machines. Machine Learning. 2002;46:389–422. doi: 10.1023/A:1012487302797

[44] Rodolfa KT, Lamba H, Ghani R. Empirical observation of negligible fairness–accuracy trade-offs in machine learning for public policy. Nature Machine Intelligence. 2021;3(10):896–904. doi: 10.1038/s42256-021-00396-x

[45] Toledo L, Jessica R, Knorst K, Ruffo F, Thiago O, Ardenghi M, et al. Machine learning in the diagnosis and prognostic prediction of dental caries: a systematic review. Caries Research. 2022 11;56:161–70. doi: 10.1159/000524167 35636386

[46] Fontana M, Carrasco-Labra A, Spallek H, Eckert G, Katz B. Improving caries risk prediction modeling: a call for action. Journal of Dental Research. 2020 10;99:1215. doi: 10.1177/0022034520934808 32600174 PMC7649255

[47] Petersen PE. Challenges to improvement of oral health in the 21st century—The approach of the WHO Global Oral Health Programme. International Dental Journal. 2004;54:329–43. doi: 10.1111/j.1875-595X.2004.tb00009.x 15631094

[48] Elani HW, Batista AFM, Thomson WM, Kawachi I, Chiavegatto Filho ADP. Predictors of tooth loss: a machine learning approach. PLOS ONE. 2021;16(6):1–14. Available from: 10.1371/journal.pone.0252873. 34143814 PMC8213149

[49] Geleto A, Sinba E, Ali MM. Dental caries and associated factors among patients visiting Shashamane Comprehensive Specialized Hospital. PLOS ONE. 2022;17(3):1–10. doi: 10.1371/journal.pone.0265000 35239749 PMC8893641

[50] Pinto-Sarmento TCdA, Abreu MH, Gomes MC, Costa EMMdB, Martins CC, Granville-Garcia AF, et al. Determinant factors of untreated dental caries and lesion activity in preschool children using ICDAS. PLOS ONE. 2016;11(2):1–11. doi: 10.1371/journal.pone.0150116 26900846 PMC4763475

[51] Powell LV. Caries prediction: a review of the literature. Community Dentistry and Oral Epidemiology. 1998;26(6):361–71. doi: 10.1111/j.1600-0528.1998.tb01974.x 9870535

[52] Christodoulou E, Ma J, Collins GS, Steyerberg EW, Verbakel JY, Calster BV. A systematic review shows no performance benefit of machine learning over logistic regression for clinical prediction models. Journal of Clinical Epidemiology. 2019 6;110:12–22. doi: 10.1016/j.jclinepi.2019.02.004 30763612

[53] Featherstone J, Doméjean S, Jenson L, Wolff M, Young D. Caries risk assessment in practice for age 6 through adult. Journal of the California Dental Association. 2007;35:703–7, 710. doi: 10.1080/19424396.2007.12221276 18044378

[54] Chaffee BW, Cheng J, Featherstone JD. Baseline caries risk assessment as a predictor of caries incidence. Journal of dentistry. 2015;43(5):518–24. doi: 10.1016/j.jdent.2015.02.013 25731155 PMC4417378

[55] Hänsel Petersson G, Isberg PE, Twetman S. Caries risk assessment in school children using a reduced Cariogram model without saliva tests. BMC oral health. 2010;10:5. doi: 10.1186/1472-6831-10-5 20403163 PMC2864191

[56] da Cunha IP, de Lacerda VR, da Silveira Gaspar G, de Lucena EHG, Mialhe FL, de Goes PSA, et al. Factors associated with the absence of Brazilians in specialized dental centers. BMC Oral Health. 2022 12;22:1–10. doi: 10.1186/s12903-022-02402-z 36028829 PMC9419406

[57] Rudin C. Stop explaining black box machine learning models for high stakes decisions and use interpretable models instead. Nature Machine Intelligence. 2019;1(5):206–15. doi: 10.1038/s42256-019-0048-x 35603010 PMC9122117

[58] Galvão MHR, Roncalli AG. Does the implementation of a national oral health policy reduce inequalities in oral health services utilization? The Brazilian experience. BMC Public Health. 2021;21(1):1–8. doi: 10.1186/s12889-021-10586-2 33740941 PMC7980604

[59] Molarius A, Engström S, Flink H, Simonsson B, Tegelberg Å. Socioeconomic differences in self-rated oral health and dental care utilisation after the dental care reform in 2008 in Sweden. BMC Oral Health. 2014;14(1):1–8. doi: 10.1186/1472-6831-14-134 25403781 PMC4240880

[60] Fonseca EPD, Frias AC, Mialhe FL, Pereira AC, Meneghim M de C. Factors associated with last dental visit or not to visit the dentist by Brazilian adolescents: a population-based study. PLoS ONE. 2017 8;12:e0183310. doi: 10.1371/journal.pone.0183310 28859102 PMC5578480

[61] Halasa-Rappel YA, Ng MW, Gaumer G, Banks DA. How useful are current caries risk assessment tools in informing the oral health care decision-making process? The Journal of the American Dental Association. 2019 2;150:91–102.e2. doi: 10.1016/j.adaj.2018.11.011 30691581

[62] Damschroder LJ, Aron DC, Keith RE, Kirsh SR, Alexander JA, Lowery JC. Fostering implementation of health services research findings into practice: A consolidated framework for advancing implementation science. Implementation Science. 2009 8;4:1–15. doi: 10.1186/1748-5908-4-50 19664226 PMC2736161

