# Supplementary material for: Low-cost and scalable machine learning model for identifying children and adolescents with poor oral health using survey data: An empirical study in Portugal
Source: PLoS One. 2025 Jan 24;20(1):e0312075. doi: 10.1371/journal.pone.0312075 (PMC11759376; doi:10.1371/journal.pone.0312075)
Supplement: S1 Table — (DOCX) [file pone.0312075.s001.docx]

Characterization of the numeric variables included in the project

| Variable | Description | Min. | Max. | Mean (SD) | Frequency missing (n) | % missing |
| --- | --- | --- | --- | --- | --- | --- |
| DMFT | Decayed, missing and filled index of each student (highest value between dmft and DMFT for students with both dentitions) | 0 | 19 | 2.17 (2.62) | 0 | 0.00 |
| Age | Students’ age, in years | 5 | 22 | 10.97 (3.08) | 0 | 0.00 |
| OftenBrushesWeek | How often does the student brushes his/her teeth, per week (1=Rarely, 2= 2 or 3 times per week, 3= 4 or 5 times per week, 4=Every day) | 1 | 4 | 1.16 (0.55) | 24 | 1.13 |
| OftenBrushesDay | How often does the student brushes his/her teeth, per day | 1 | 5 | 2.40 (0.87) | 214 | 10.03 |
| HowFrequentlyDentist | How frequently does the student visit the dentist (1 – less than once per year, 2 – once per year, 3 – more than once per year) | 1 | 3 | 2.29 (0.69) | 558 | 26.16 |
| OftenShouldBrush | How often does the student believe people should brush their teeth per day (1 = once, 2 = twice, 3 = three times, 4 = four times, 5 = five times) | 1 | 5 | 3.26 (0.82) | 52 | 2.44 |
| ShouldBrushHowLong | How long should the brushing take? (1 = 1 min., 2 = 3 min., 3 = 5 min.) | 1 | 3 | 2.21 (0.58) | 66 | 3.09 |
| MealsPerDay | How many meals the student has per day | 1 | 8 | 4.59 (1.11) | 33 | 1.55 |
| Breakfast | If the student usually takes breakfast (1 = Never, 2 = Rarely, 3 = Sometimes, 4 = Always) | 1 | 4 | 3.76(0.55 | 25 | 1.17 |
| EatsDinner | If the student typically has dinner (1 = Always, 2 = Sometimes, 3 = Rarely, 4 = Never) | 1 | 4 | 1.05 (0.22) | 631 | 29.58 |
| GoesRestaurant | If the student’s family often goes to a restaurant (1 = Never, 2 = Rarely, 3 = Some weekends, 4 = Every weekend, 5 = During the week) | 1 | 5 | 2.39 (0.76) | 87 | 4.08 |
| CupsWater | How many cups of water does the student drink per day (1 = rarely drink, 2 = 1 to 2, 3 = 3 to 4, 4 = 5 to 6, 5 = 7 or more) | 1 | 5 | 3.47 (1.20) | 610 | 28.60 |
| SugaryBreakfast | How many distinct sugary food items the student has for breakfast | 0 | 8 | 1.14 (1.40) | 0 | 0.0 |
| SugaryMorningSnack | How many distinct sugary food items the student has for morning snack | 0 | 10 | 1.01 (1.25) | 0 | 0.0 |
| SugaryLunch | How many distinct sugary food items the student typically has for lunch | 0 | 3 | 0.48 (0.69) | 0 | 0.0 |
| SugaryAfternoonSnack | How many distinct sugary food items the student typically has for afternoon snack | 0 | 9 | 1.37 (1.49) | 0 | 0.0 |
| SugaryBeforeDinner | How many distinct sugary food items the student typically has before dinner | 0 | 10 | 0.67 (1.22) | 0 | 0.0 |
| SugaryDinner | How many distinct sugary food items the student typically has for dinner | 0 | 3 | 0.46 (0.70) | 0 | 0.0 |
| SugaryAfterDinner | How many distinct sugary food items the student typically has after dinner | 0 | 9 | 0.48 (1.00) | 0 | 0.0 |
| SugaryTotal | Sum of how many distinct sugary food items the student typically has during the day | 0 | 53 | 5.60  (5.46) | 0 | 0.0 |
| DairyBreakfast | How many distinct dairy food items the student has for breakfast | 0 | 3 | 1.68 (0.80) | 0 | 0.0 |
| DairyMorningSnack | How many distinct dairy food items the student has for morning snack | 0 | 3 | 1.01 (0.95) | 0 | 0.0 |
| DairyAfternoonSnack | How many distinct dairy food items the student typically has for afternoon snack | 0 | 3 | 1.46 (0.99) | 0 | 0.0 |
| DairyBeforeDinner | How many distinct dairy food items the student typically has before dinner | 0 | 3 | 0.53 (0.83) | 0 | 0.0 |
| DairyAfterDinner | How many distinct dairy food items the student typically has after dinner | 0 | 3 | 0.69 (0.76) | 0 | 0.0 |
| DairyTotal | Sum of how many distinct dairy food items the student typically has during the day | 0 | 15 | 5.37 (2.88) | 0 | 0.0 |
| SaltyBreakfast | How many distinct salty fast-food items the student has for breakfast | 0 | 3 | 0.19 (0.45) | 0 | 0.0 |
| SaltyMorningSnack | How many distinct salty fast-food items the student has for morning snack | 0 | 3 | 0.24 (0.50) | 0 | 0.0 |
| SaltyLunch | How many distinct salty fast-food items the student typically has for lunch | 0 | 4 | 0.61 (0.96) | 0 | 0.0 |
| SaltyAfternoonSnack | How many distinct salty fast-food items the student typically has for afternoon snack | 0 | 3 | 0.27 (0.54) | 0 | 0.0 |
| SaltyBeforeDinner | How many distinct salty fast-food items the student typically has before dinner | 0 | 3 | 0.17 (0.45) | 0 | 0.0 |
| SaltyDinner | How many distinct salty fast-food items the student typically has for dinner | 0 | 4 | 0.68 (1.00) | 0 | 0.0 |
| SaltyAfterDinner | How many distinct salty fast-food items the student typically has after dinner | 0 | 3 | 0.10 (0.33) | 0 | 0.0 |
| SaltyTotal | Sum of how many distinct salty fast-food items the student typically has during the day | 0 | 22 | 2.25 (2.80) | 0 | 0.0 |
| HealthyBreakfast | How many distinct healthy food items the student has for breakfast | 0 | 2 | 0.39 (0.59) | 0 | 0.0 |
| HealthyMorningSnack | How many distinct healthy food items the student has for morning snack | 0 | 2 | 0.29 (0.47) | 0 | 0.0 |
| HealthyLunch | How many distinct healthy food items the student typically has for lunch | 0 | 5 | 2.94 (1.56) | 0 | 0.0 |
| HealthyAfternoonSnack | How many distinct healthy food items the student typically has for afternoon snack | 0 | 2 | 0.39 (0.55) | 0 | 0.0 |
| HealthyBeforeDinner | How many distinct healthy food items the student typically has before dinner | 0 | 2 | 0.25 (0.47) | 0 | 0.0 |
| HealthyDinner | How many distinct healthy food items the student typically has for dinner | 0 | 5 | 2.78 (1.47) | 0 | 0.0 |
| HealthyAfterDinner | How many distinct healthy food items the student typically has after dinner | 0 | 2 | 0.18 (0.44) | 0 | 0.0 |
| HealthyTotal | Sum of how many distinct healthy food items the student typically has during the day | 0 | 19 | 7.22 (3.68) | 0 | 0.0 |
| SportsExtraSchool | Whether the student does sports after school (1 = never or less than once a week, 2 = once a week, 3 = 2 to 3 times a week, 4 = almost every day) | 1 | 4 | 2.39 (1.18) | 153 | 7.17 |
| LeisureActivities | If the student takes part in leisurely activities (1 = never or less than once a week, 2 = once a week, 3 = 2 to 3 times a week, 4 = almost every day) | 1 | 4 | 2.19 (1.17) | 240 | 11.25 |
| Sports20min | How often does the student does sport activities for at least 20 minutes, outside school (1 = Never or less than once per month, 2 = 2 to 4 times per month, 3 = 2 to 3 times per week, 4 = 4 to 6 times per week, 5 = every day) | 1 | 5 | 2.69 (1.28) | 168 | 7.88 |
| SportsLooseBreath | How long the student does sports vigorously enough to loose breath or sweat outside school, per week (1 = never, 2 = 30 minutes to 1 hour, 3 = 2 to 3 hours, 4 = 4 to 6 hours, 5 = 7 or more hours) | 1 | 5 | 2.17 (1.00) | 144 | 6.75 |

Note: Total n = 2133
