# Supplementary material for: Low-cost and scalable machine learning model for identifying children and adolescents with poor oral health using survey data: An empirical study in Portugal
Source: PLoS One. 2025 Jan 24;20(1):e0312075. doi: 10.1371/journal.pone.0312075 (PMC11759376; doi:10.1371/journal.pone.0312075)
Supplement: S2 Table — (DOCX) [file pone.0312075.s002.docx]

Characterization of the binary variables included in the project

| Variable | Description | Values | Frequency (n) | Percentage (%) | Frequency missing (n) | % missing |
| --- | --- | --- | --- | --- | --- | --- |
| Gender | If the student is female | Yes | 1017 | 47.68 | 0 | 0.00 |
|  |  | No | 1116 | 52.32 |  |  |
| SocialAssistanceA | If the student receives the highest level of social assistance (level A) | Yes | 432 | 20.25 | 0 | 0.00 |
|  |  | No | 1701 | 79.75 |  |  |
| SocialAssistanceB | If the student receives the lowest level of social assistance (level B) | Yes | 258 | 12.10 | 0 | 0.00 |
|  |  | No | 1875 | 87.90 |  |  |
| BrushesTeeth | If the student typically brushes his/hers teeth | Yes | 2111 | 98.97 | 14 | 0.66 |
|  |  | No | 8 | 0.38 |  |  |
| BrushBeforeMeals | If the student typically brushes his/her teeth before meals | Yes | 153 | 7.17 | 46 | 2.16 |
|  |  | No | 1934 | 90.67 |  |  |
| BrushAfterMeals | If the student typically brushes his/her teeth after meals | Yes | 1616 | 75.76 | 46 | 2.16 |
|  |  | No | 471 | 22.08 |  |  |
| Howlongbrushes1min | If the student typically brushes his/her teeth for one minute | Yes | 338 | 15.85 | 41 | 1.92 |
|  |  | No | 1795 | 84.15 |  |  |
| Howlongbrushes3min | If the student typically brushes his/her teeth for three minutes | Yes | 876 | 41.07 | 41 | 1.92 |
|  |  | No | 1257 | 58.93 |  |  |
| Howlongbrushes5min | If the student typically brushes his/her teeth for five minutes | Yes | 261 | 12.24 | 41 | 1.92 |
|  |  | No | 1872 | 87.76 |  |  |
| BrushSchool | If the student typically brushes his/her teeth at school | Yes | 89 | 4.17 | 38 | 1.78 |
|  |  | No | 2006 | 94.05 |  |  |
| BrushAdult | If the student typically brushes his/her teeth together with an adult, at school | Yes | 39 | 1.83 | 832 | 39.01 |
|  |  | No | 1262 | 59.17 |  |  |
| BrushBeforeBed | If the student typically brushes his/her teeth before bed | Yes | 1919 | 89.97 | 41 | 1.92 |
|  |  | No | 173 | 8.11 |  |  |
| Floss | If the student typically flosses | Yes | 504 | 23.63 | 50 | 2.34 |
|  |  | No | 1579 | 74.03 |  |  |
| MouthWash | If the student typically uses mouth wash | Yes | 1173 | 54.99 | 110 | 5.16 |
|  |  | No | 850 | 39.85 |  |  |
| GoesDentist | If the student frequently visits the dentist | Yes | 1602 | 75.11 | 51 | 2.39 |
|  |  | No | 480 | 22.50 |  |  |
| ImportantBrush | If the student considers important to brush teeth | Yes | 2100 | 98.45 | 32 | 1.50 |
|  |  | No | 1 | 0.05 |  |  |
| WhotaughtParent | If the student was taught to brush their teeth by a parent | Yes | 1646 | 77.17 | 349 | 16.36 |
|  |  | No | 138 | 6.47 |  |  |
| WhotaughtProfessor | If the student was taught to brush their teeth by a professor/educator | Yes | 10 | 0.47 | 349 | 16.36 |
|  |  | No | 1774 | 83.17 |  |  |
| WhotaughtH ygienist | If the student was taught to brush their teeth by an oral hygienist | Yes | 15 | 0.70 | 349 | 16.36 |
|  |  | No | 1769 | 82.93 |  |  |
| WhotaughtDentist | If the student was taught to brush their teeth by a dentist | Yes | 50 | 2.34 | 349 | 16.36 |
|  |  | No | 1734 | 81.29 |  |  |
| ShouldBrushBefore | If the student believe people should brush their teeth before meals | Yes | 85 | 3.98 | 50 | 2.34 |
|  |  | No | 1998 | 93.67 |  |  |
| ShouldBrushAfter | If the student believe people should brush their teeth after meals | Yes | 1807 | 84.72 | 50 | 2.34 |
|  |  | No | 276 | 12.94 |  |  |
| KnowsFloss | If the student knows how to floss | Yes | 1366 | 64.04 | 76 | 3.56 |
|  |  | No | 691 | 32.40 |  |  |
| ShoudlFlossBefore | If the student believes the best time to floss is before brushing teeth | Yes | 406 | 19.03 | 312 | 14.63 |
|  |  | No | 1415 | 66.34 |  |  |
| ShouldFlossAfter | If the student believes the best time to floss is after brushing teeth | Yes | 1286 | 60.29 | 312 | 14.63 |
|  |  | No | 535 | 25.08 |  |  |
| HeardCaries | If the student ever heard about a disease called Caries | Yes | 1989 | 93.25 | 0 | 0.00 |
|  |  | No | 144 | 6.75 |  |  |
| HeardBacterial | If the student ever heard about a disease called Bacterial Plaque | Yes | 1362 | 63.85 | 0 | 0.00 |
|  |  | No | 771 | 36.15 |  |  |
| HeardTartar | If the student ever heard about a disease called Tartar | Yes | 938 | 43.98 | 0 | 0.00 |
|  |  | No | 1195 | 56.02 |  |  |
| HeardGengivitis | If the student ever heard about a disease called Gengivitis | Yes | 1207 | 56.59 | 0 | 0.00 |
|  |  | No | 926 | 43.41 |  |  |
| HeardPeriodontitis | If the student ever heard about a disease called Periodontitis | Yes | 139 | 6.52 | 0 | 0.00 |
|  |  | No | 1994 | 93.48 |  |  |
| ShouldUseFloss | If the student believes that besides a toothbrush, people should use floss to clean their teeth | Yes | 1741 | 81.62 | 0 | 0.00 |
|  |  | No | 392 | 18.38 |  |  |
| ShouldUseBrush | If the student belies that besides a toothbrush, people should use other brushes to clean their teeth | Yes | 344 | 16.13 | 0 | 0.00 |
|  |  | No | 1789 | 83.87 |  |  |
| ShouldUseSugarlessGum | If the student belies that besides a toothbrush, people should use sugarless gum to clean their teeth | Yes | 129 | 6.05 | 0 | 0.00 |
|  |  | No | 2004 | 93.95 |  |  |
| ShouldUseMouthWash | If the student belies that besides a toothbrush, people should use mouth wash to clean their teeth | Yes | 1738 | 81.48 | 0 | 0.00 |
|  |  | No | 395 | 18.52 |  |  |
| ShouldUseToothPaste | If the student belies that besides a toothbrush, people should use toothpaste to clean their teeth | Yes | 1905 | 89.31 | 0 | 0.00 |
|  |  | No | 228 | 10.69 |  |  |
| ShouldUseWater | If the student belies that besides a toothbrush, people should use water to clean their teeth | Yes | 1334 | 62.54 | 0 | 0.00 |
|  |  | No | 799 | 37.46 |  |  |
| ShouldUseToothPicks | If the student belies that besides a toothbrush, people should use toothpicks to clean their teeth | Yes | 80 | 3.75 | 0 | 0.00 |
|  |  | No | 2053 | 96.25 |  |  |
| ShouldUseApple | If the student belies that besides a toothbrush, people should use apples to clean their teeth | Yes | 145 | 6.80 | 0 | 0.00 |
|  |  | No | 1988 | 93.20 |  |  |
| LunchHome | If the student typically has lunch at home | Yes | 934 | 43.79 | 0 | 0.00 |
|  |  | No | 1199 | 56.21 |  |  |
| LunchCanteen | If the student typically has lunch at the school canteen | Yes | 871 | 40.83 | 0 | 0.00 |
|  |  | No | 1262 | 59.17 |  |  |
| LunchBar | If the student typically has lunch at the school bar | Yes | 11 | 0.52 | 0 | 0.00 |
|  |  | No | 2122 | 99.48 |  |  |
| EatsBeforeDinner | If the student typically eats or drinks between afternoon snack and dinner | Yes | 1266 | 59.35 | 86 | 4.03 |
|  |  | No | 781 | 36.62 |  |  |
| EatsBeforeBed | If the student typically eats or drinks before bed | Yes | 1289 | 60.43 | 70 | 3.28 |
|  |  | No | 774 | 36.29 |  |  |
| WaterLunch | If the student typically drinks water at lunch | Yes | 1197 | 56.12 | 0 | 0.0 |
|  |  | No | 936 | 43.88 |  |  |
| WaterDinner | If the student typically drinks water at dinner | Yes | 1053 | 49.37 | 0 | 0.0 |
|  |  | No | 1080 | 50.63 |  |  |
| SportsCompetitionsNever | If the student never participated in sports competitions | Yes | 726 | 34.04 | 227 | 10.64 |
|  |  | No | 1180 | 55.32 |  |  |
| SportsCompetitionsPast | If the student participated in sports competitions in the past | Yes | 478 | 22.41 | 227 | 10.64 |
|  |  | No | 1428 | 66.95 |  |  |
| SportsCompetitionsInterschool | If the student participates in sports competitions at the interschool level | Yes | 269 | 12.61 | 227 | 10.64 |
|  |  | No | 1637 | 76.75 |  |  |
| SportCompetitionsClub | If the student participates in sports competitions at a club level | Yes | 370 | 17.35 | 227 | 10.64 |
|  |  | No | 1536 | 72.01 |  |  |

Note: Total n = 2133
