# Supplementary material for: Low-cost and scalable machine learning model for identifying children and adolescents with poor oral health using survey data: An empirical study in Portugal
Source: PLoS One. 2025 Jan 24;20(1):e0312075. doi: 10.1371/journal.pone.0312075 (PMC11759376; doi:10.1371/journal.pone.0312075)
Supplement: S3 Table — (DOCX) [file pone.0312075.s003.docx]

Food items classification

The following table contains transformations of individual food items into more informative food categories. As such, we split the existing food items into categories that were deemed informative from an oral health point of view. Water was labelled as healthy, but we included a separate count for the quantity of water. We requested assistance from a dietician of the partner organization of this research project to validate our approach.

*Table 6: food items classification*

| Food Item | Processed Sugary | Dairy | Processed Salty | Healthy |
| --- | --- | --- | --- | --- |
| Yogurt | x | x |  |  |
| Milk |  | x |  |  |
| Chocolate Powder | x |  |  |  |
| Natural Cereals |  |  |  |  |
| Sugared Cereals | x |  |  |  |
| Tea |  |  |  | x |
| Coffee |  |  |  |  |
| Sugar | x |  |  |  |
| Natural Juices |  |  |  |  |
| Packaged Juices |  |  |  |  |
| Sodas | x |  |  |  |
| Bread |  |  |  |  |
| Butter/Margarine |  | x |  |  |
| Cheese/Ham |  |  |  |  |
| Spread/Jam | x |  |  |  |
| Fuit |  |  |  | x |
| Pastries | x |  |  |  |
| Homemade Pastries | x |  |  |  |
| Packaged Pastries (Donuts etc.) | x |  |  |  |
| Cookies | x |  |  |  |
| Salted Cookies |  |  | x |  |
| Salty Pastries |  |  | x |  |
| Fried Packaged Items (Fries) |  |  | x |  |
| Sweets | x |  |  |  |
| Soup |  |  |  | x |
| Meat/Fish |  |  |  |  |
| Pasta/Rice/Potatoe |  |  |  |  |
| Beans/Chickpeas |  |  |  | x |
| Legumes/Salad |  |  |  | x |
| Open Sandwich |  |  | x |  |
| Pizza, Hamburger |  |  | x |  |
| Sweet Desert | x |  |  |  |
| Water* |  |  |  | x |

* Note: Water was included as a separate food category in the variable matrix.
