# Supplementary material for: Low-cost and scalable machine learning model for identifying children and adolescents with poor oral health using survey data: An empirical study in Portugal
Source: PLoS One. 2025 Jan 24;20(1):e0312075. doi: 10.1371/journal.pone.0312075 (PMC11759376; doi:10.1371/journal.pone.0312075)
Supplement: S4 Table — (DOCX) [file pone.0312075.s004.docx]

Best models detailed characteristics

*Table 7: Best models detailed characteristics*

| Characteristics | DMFT3 Model | DMFT4 Model |
| --- | --- | --- |
| Algorithm | Logistic Regression | Logistic Regression |
| Selected Number of Features | 17 | 12 |
| Regularization Strength (C) | 0.005 | 0.01 |
| Penalty | L2 | L2 |
| Solver | LBFGS | LBFGS |
| Categorical Low-Variance Threshold | 0.5 | 0.05 |
| Numerical Low-Variance Threshold | 0 | 0 |
| Imputation Strategy | Mean | Mean |

Note: To find the best combinations of characteristics of the models, we tuned the algorithms’ hyperparameters using grid search.
