# Supplementary figures and images for: Low-cost and scalable machine learning model for identifying children and adolescents with poor oral health using survey data: An empirical study in Portugal
Source: PLoS One. 2025 Jan 24;20(1):e0312075. doi: 10.1371/journal.pone.0312075 (PMC11759376; doi:10.1371/journal.pone.0312075)

ROC AUC curves for DMFT3 and DMFT4


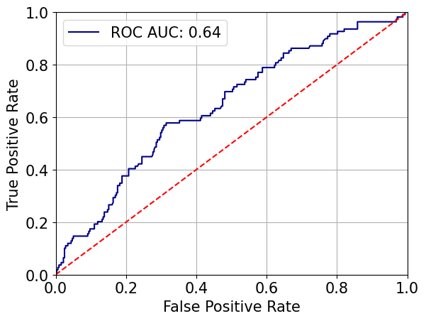

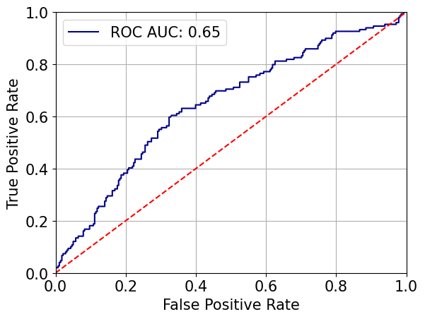


*Figure 8: ROC AUC curves for DMFT3 (left panel) and DMFT4 (right panel)*

Supplement: S1 Fig — (DOCX) [file pone.0312075.s005.docx]
