# Supplementary material for: Low-cost and scalable machine learning model for identifying children and adolescents with poor oral health using survey data: An empirical study in Portugal
Source: PLoS One. 2025 Jan 24;20(1):e0312075. doi: 10.1371/journal.pone.0312075 (PMC11759376; doi:10.1371/journal.pone.0312075)
Supplement: S1 File — (DOCX) [file pone.0312075.s006.docx]

Detailed description of the experts’ model

To create a rule-based model representing what would be the selection of students needing a dental appointment based solely in experts’ knowledge and in the absence of a machine learning model, we created a short questionnaire asking APPSHO’s dentists: Q1) whether a given variable was relevant to predict whether the student is in-need or not, from a list of 10 variables that are typically associated with the DMFT index; Q2) whether each of the selected factors in Q1 would increase or decrease the probability of a student being in-need; and Q3) the relative weight of each of the selected variables, in a scale ranging from 1 – Less important to 10 – More important. From the answers to these questions, we calculated the weights for each of the 10 factors. The higher the absolute value, the more importance attributed by the dentists to the factors; factors with negative values reduce the risk of having a high DMFT index, and factors with positive values increase the risk. The factors and the weights are presented below, ordered by absolute weight.

*Table 5: factors and weights of the expert model*

| Factor | Weight |
| --- | --- |
| Sugary foods intake | 9.125 |
| Brush teeth 3x | -9.000 |
| Visit dentist once a year or more | -8.625 |
| Floss | -8.500 |
| Brush teeth at school | -5.000 |
| Use mouthwash | -3.875 |
| Always eat breakfast | -3.375 |
| Eat 4-5 meals per day | -3.375 |
| Drink 2+ glasses of water | -3.000 |
| Receive social support | -2.000 |

Besides the described factors, we also included age into the risk calculation, as this prove to be a very significant factor in predicting risk. We calculated the risk of an individual student i by summing the weights of each factor j by the value the student i had in that factor, using the following equation (1):

𝑅𝑖𝑠𝑘𝑆𝑡𝑢𝑑𝑒𝑛𝑡𝑖= ∑𝑗 𝑤𝑒𝑖𝑔ℎ𝑡𝑗∗ 𝑣𝑎𝑙𝑢𝑒𝑗𝑖 (1)

Where weightj is the weight of each factor and valueji is the value of the factor for student i.
