# Supplementary material for: Low-cost and scalable machine learning model for identifying children and adolescents with poor oral health using survey data: An empirical study in Portugal
Source: PLoS One. 2025 Jan 24;20(1):e0312075. doi: 10.1371/journal.pone.0312075 (PMC11759376; doi:10.1371/journal.pone.0312075)

Figure S2.1. Distribution of students per dmft (deciduous teeth), for students younger than 12.


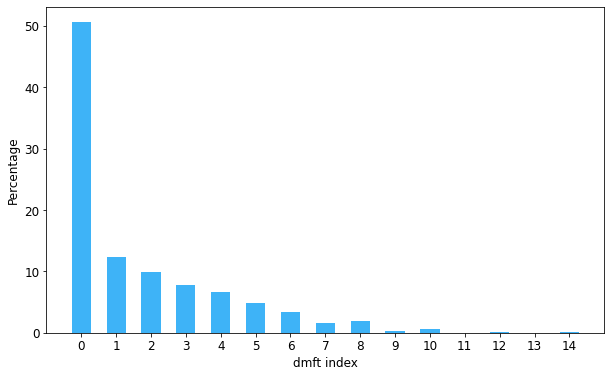


Figure S2.2. Distribution of students per DMFT (permanent teeth), for students younger than 12.


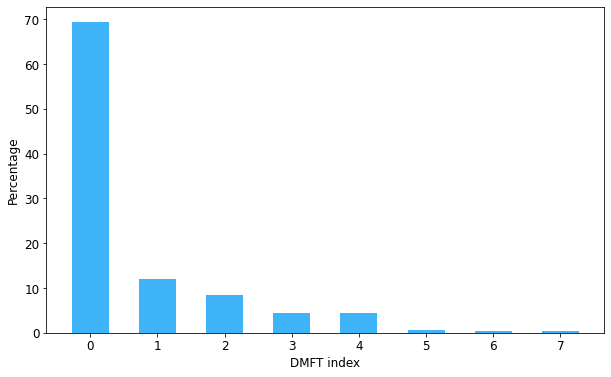


Figure S2.3. Distribution of students per DMFT (permanent teeth), for students 12 or older.


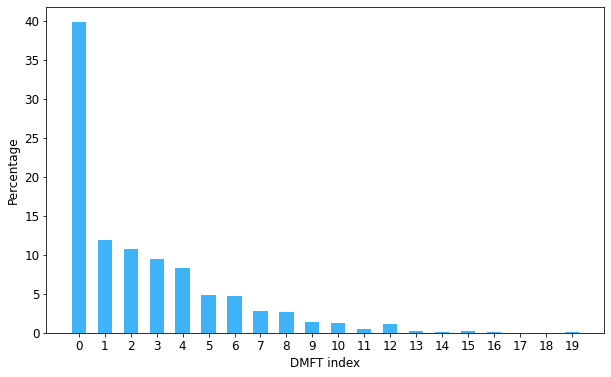

Supplement: S2 File — (DOCX) [file pone.0312075.s007.docx]
