# Supplementary material for: Low-cost and scalable machine learning model for identifying children and adolescents with poor oral health using survey data: An empirical study in Portugal
Source: PLoS One. 2025 Jan 24;20(1):e0312075. doi: 10.1371/journal.pone.0312075 (PMC11759376; doi:10.1371/journal.pone.0312075)
Supplement: S3 File — (DOCX) [file pone.0312075.s008.docx]

**Section 1. Students younger than 12 – dmft (decidous teeth)**

| Variable | Value | dmft  mean(standard deviation) | t-test p-value |
| --- | --- | --- | --- |
| Floss | Yes | 1.27(2.11) | *p = 0.003* |
|  | No | 1.74(2.32) |  |
| GoesDentist | Yes | 1.62(2.27) | *p = 0.651* |
|  | No | 1.69(2.32) |  |
| BrushSchool | Yes | 1.39(2.01) | *p = 0.312* |
|  | No | 1.66(2.31) |  |
| OftenBrushesDay | One or two times (1) | 1.68(2.29) | *(1)(2) p = 0.009*  *(1)(3) p = 0.354*  *(2)(3) p = 0.452* |
|  | Three times (2) | 1.25(2.14) |  |
|  | Four or five times (3) | 1.45(2.02) |  |
| EatsBreakfast | Always | 1.63(2.31) | *p = 0.785* |
|  | Not always | 1.58(1.93) |  |
| MealsPerDay | Less than 4 (1) | 1.87(2.34) | *(1)(2) p = 0.223* |
|  | 4-6 (2) | 1.61(2.29) | *(1)(3) p = 0.996* |
|  | More than 6 (3) | 1.86(2.06) | *(2)(3) p = 0.497* |
| SportsExtraSchool | Yes | 1.41(2.10) | *p < 0.001* |
|  | No | 2.00(2.48) |  |
| Gender | Male | 1.74(2.36) | *p = 0.136* |
|  | Female | 1.55(2.22) |  |
| Social Assistance | No assistance (1) | 1.44(2.23) | *(1)(2) p = 0.006* |
|  | Medium (2) | 1.98(2.30) | *(1)(3) p < 0.001* |
|  | High (3) | 2.06(2.37) | *(2)(3) p = 0.709* |

Table 1.1. Association between oral health, nutrition and physical activity behaviors and dmft, for students younger than 12.

|  | | Sugary | | Dairy | | Salty fast-food | | Healthy | |
| --- | --- | --- | --- | --- | --- | --- | --- | --- | --- |
|  |  | mean (standard deviation) | t-test p-value | mean (standard deviation) | t-test p-value | mean (standard deviation) | t-test p-value | mean (standard deviation) | t-test p-value |
| dmft >=3 | Yes | 5.53(5.57) | *p<0.001* | 6.23(3.18) | *p<0.001* | 2.18(2.73) | *p=0.236* | 8.37(3.73) | *p<0.001* |
|  | No | 4.51(4.05) |  | 5.47(2.65) |  | 1.98(2.52) |  | 7.51(3.60) |  |
| dmft >=4 | Yes | 5.58(5.88) | *p=0.003* | 6.14(3.26) | *p=0.004* | 2.17(2.72) | *p=0.368* | 8.35(3.83) | *p=0.004* |
|  | No | 4.60(4.12) |  | 5.56(2.69) |  | 2.00(2.54) |  | 7.60(3.60) |  |

Table 1.2. Association between dmft and distinct food items typically ingested, for students younger than 12.

**Section 2. Students younger than 12 – DMFT (permanent teeth)**

| Variable | Value | DMFT  mean(standard deviation) | t-test p-value |
| --- | --- | --- | --- |
| Floss | Yes | 0.68(1.23) | *p = 0.973* |
|  | No | 0.68(1.27) |  |
| GoesDentist | Yes | 0.68(1.27) | *p = 0.858* |
|  | No | 0.66(1.18) |  |
| BrushSchool | Yes | 0.39(0.99) | *p = 0.030* |
|  | No | 0.70(1.27) |  |
| OftenBrushesDay | One or two times (1) | 0.61(1.14) | *(1)(2) p = 0.842*  *(1)(3) p = 0.091*  *(2)(3) p = 0.210* |
|  | Three times (2) | 0.62(1.29) |  |
|  | Four or five times (3) | 0.83(1.45) |  |
| EatsBreakfast | Always | 0.63(1.19) | *p < 0.001* |
|  | Not always | 1.13(1.70) |  |
| MealsPerDay | Less than 4 (1) | 0.83(1.38) | *(1)(2) p = 0.103*  *(1)(3) p = 0.115*  *(2)(3) p = 0.002* |
|  | 4-6 (2) | 0.64(1.21) |  |
|  | More than 6 (3) | 1.27(1.85) |  |
| SportsExtraSchool | Yes | 0.64(2.28) | *p = 0.137* |
|  | No | 0.76(1.23) |  |
| Gender | Male | 0.64(1.23) | *p = 0.297* |
|  | Female | 0.71(1.28) |  |
| Social Assistance | No assistance (1) | 0.64(1.22) | *(1)(2) p = 0.097*  *(1)(3) p = 0.524*  *(2)(3) p = 0.345* |
|  | Medium (2) | 0.82(1.43) |  |
|  | High (3) | 0.70(1.21) |  |

Table 2.1. Association between oral health, nutrition and physical activity behaviors and DMFT (permanent teeth), for students younger than 12.

|  | | Sugary | | Dairy | | Salty fast-food | | Healthy | |
| --- | --- | --- | --- | --- | --- | --- | --- | --- | --- |
|  |  | mean (standard deviation) | t-test p-value | mean (standard deviation) | t-test p-value | mean (standard deviation) | t-test p-value | mean (standard deviation) | t-test p-value |
| DMFT>=3 | Yes | 5.48(5.45) | *p=0.073* | 5.73(2.86) | *p=0.809* | 2.37(2.93) | *p=0.122* | 7.56(4.15) | *p=0.536* |
|  | No | 4.71(4.41) |  | 5.66(2.82) |  | 2.00(2.54) |  | 7.77(3.59) |  |
| DMFT>=4 | Yes | 5.76(5.68) | *p=0.063* | 5.65(3.00) | *p=0.939* | 2.55(3.17) | *p=0.084* | 7.52(4.28) | *p=0.593* |
|  | No | 4.73(4.45) |  | 5.67(2.81) |  | 2.00(2.54) |  | 7.76(3.61) |  |

Table 2.2. Association between DMFT (permanent teeth) and distinct food items typically ingested in a day, for students younger than 12.

**Section 3. Students 12 or older – DMFT (permanent teeth)**

| Variable | Value | DMFT  mean (standard deviation) | t-test p-value |
| --- | --- | --- | --- |
| Floss | Yes | 2.35(2.86) | *p=0.656* |
|  | No | 2.45(3.04) |  |
| GoesDentist | Yes | 2.24(2.86) | *p=0.002* |
|  | No | 2.95(3.40) |  |
| BrushSchool | Yes | 2.44(2.70) | *p=0.995* |
|  | No | 2.44(3.02) |  |
| OftenBrushesDay | One or two times (1) | 2.56(3.13) | *(1)(2) p=0.041*  *(1)(3) p=0.811*  *(2)(3) p=0.262* |
|  | Three times (2) | 2.10(2.77) |  |
|  | Four or five times (3) | 3.14(2.48) |  |
| EatsBreakfast | Always | 2.11(2.85) | *p<0.001* |
|  | Not always | 3.05(3.22) |  |
| MealsPerDay | Less than 4 (1) | 2.85(2.17) | *(1)(2) p=0.030* |
|  | 4-6 (2) | 2.29(2.57) | *(1)(3) p=0.398* |
|  | More than 6 (3) | 2.40(2.23) | *(2)(3) p=0.775* |
| SportsExtraSchool | Yes | 2.35(2.97) | *p=0.396* |
|  | No | 2.53(3.08) |  |
| Gender | Male | 2.11(2.73) | *p=0.005* |
|  | Female | 2.68(3.20) |  |
| Social Assistance | No assistance (1) | 2.18(2.74) | *(1)(2) p=0.004* |
|  | Medium (2) | 3.08(3.64) | *(1)(3) p=0.003* |
|  | High (3) | 2.91(3.41) | *(2)(3) p=0.687* |

Table 3.1. Association between oral health, nutrition and physical activity behaviors and DMFT (permanent teeth), for students 12 or older.

|  | | Sugary | | Dairy | | Salty fast-food | | Healthy | |
| --- | --- | --- | --- | --- | --- | --- | --- | --- | --- |
|  |  | mean (standard deviation) | t-test p-value | mean (standard deviation) | t-test p-value | mean (standard deviation) | t-test p-value | mean (standard deviation) | t-test p-value |
| DMFT>=3 | Yes | 7.58(7.04) | *p=0.001* | 4.97(3.06) | *p=0.856* | 2.98(3.32) | *P<0.001* | 6.59(3.66) | *p=0.524* |
|  | No | 6.19(5.83) |  | 4.93(2.82) |  | 2.28(2.85) |  | 6.44(3.57) |  |
| DMFT>=4 | Yes | 7.30(7.06) | *p=0.078* | 4.91(2.98) | *p=0.804* | 2.89(3.20) | *p=0.033* | 6.43(3.60) | *p=0.733* |
|  | No | 6.48(6.03) |  | 4.96(2.88) |  | 2.40(2.98) |  | 6.52(3.60) |  |

Table 3.2. Association between DMFT (permanent teeth) and distinct food items typically ingested, for students 12 or older.
