# Supplementary material for: Low-cost and scalable machine learning model for identifying children and adolescents with poor oral health using survey data: An empirical study in Portugal
Source: PLoS One. 2025 Jan 24;20(1):e0312075. doi: 10.1371/journal.pone.0312075 (PMC11759376; doi:10.1371/journal.pone.0312075)
Supplement: S4 File — (DOCX) [file pone.0312075.s009.docx]

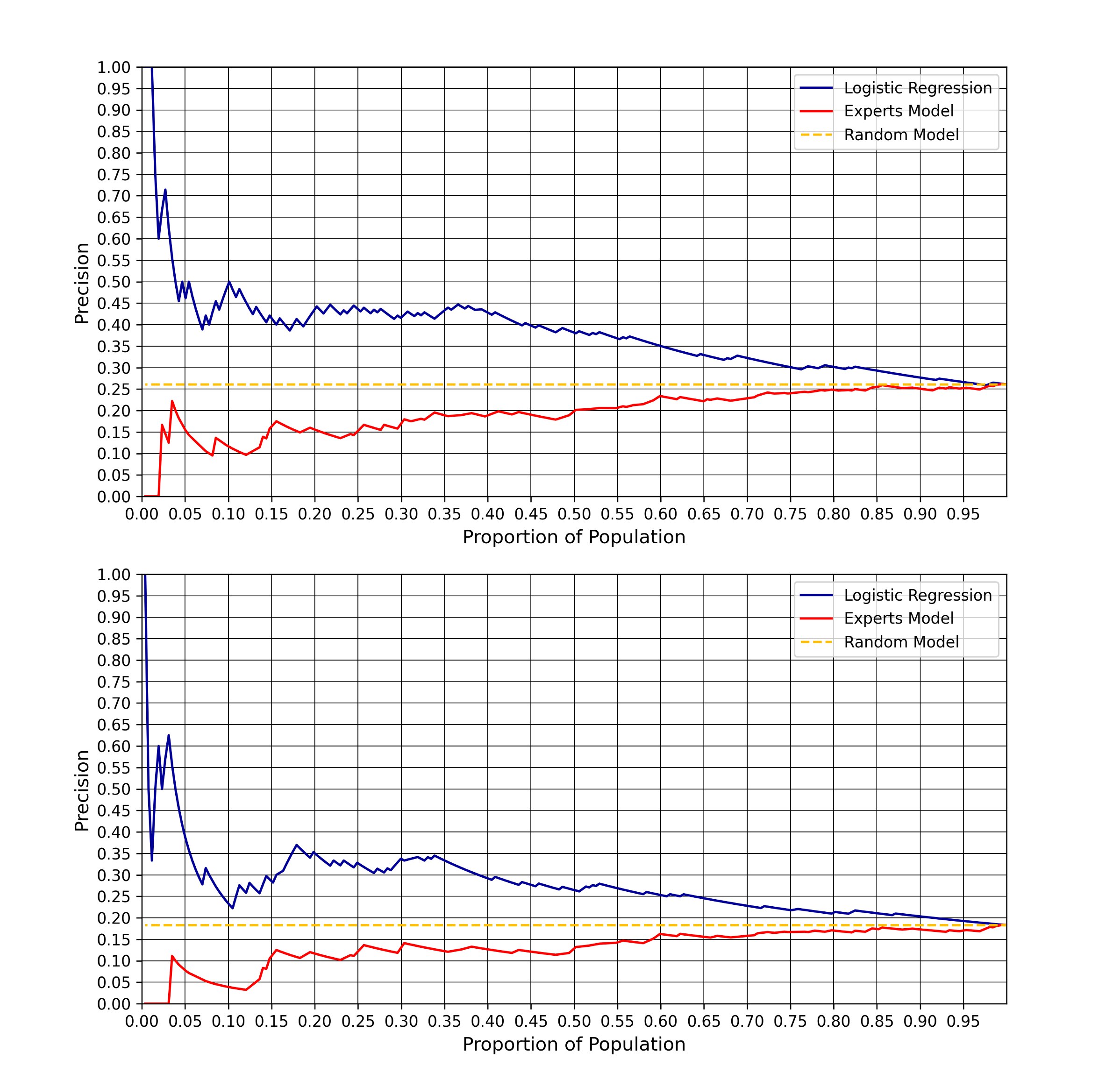


Figure S4.1. Precision curves for the DMFT3 model (above) and the DMFT4 model (below) comparing with both the experts and the random model, for the test subsample of students younger than 12, using dmft (deciduous teeth) as the target variable.


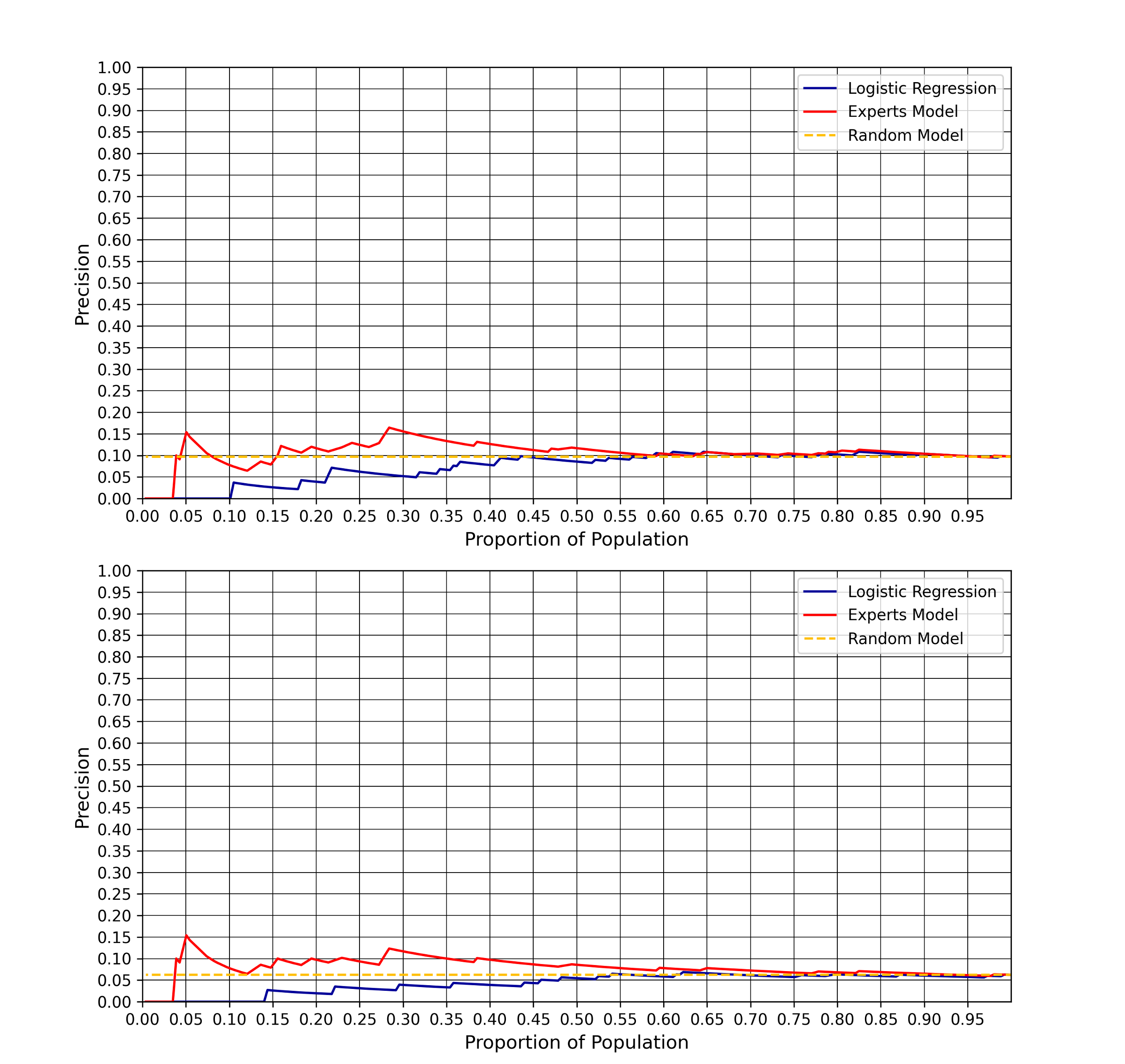


Figure S4.2. Precision curves for the DMFT3 model (above) and the DMFT4 model (below) comparing with both the experts and the random model, for the test subsample of students younger than 12, using DMFT (permanent teeth) as the target variable.


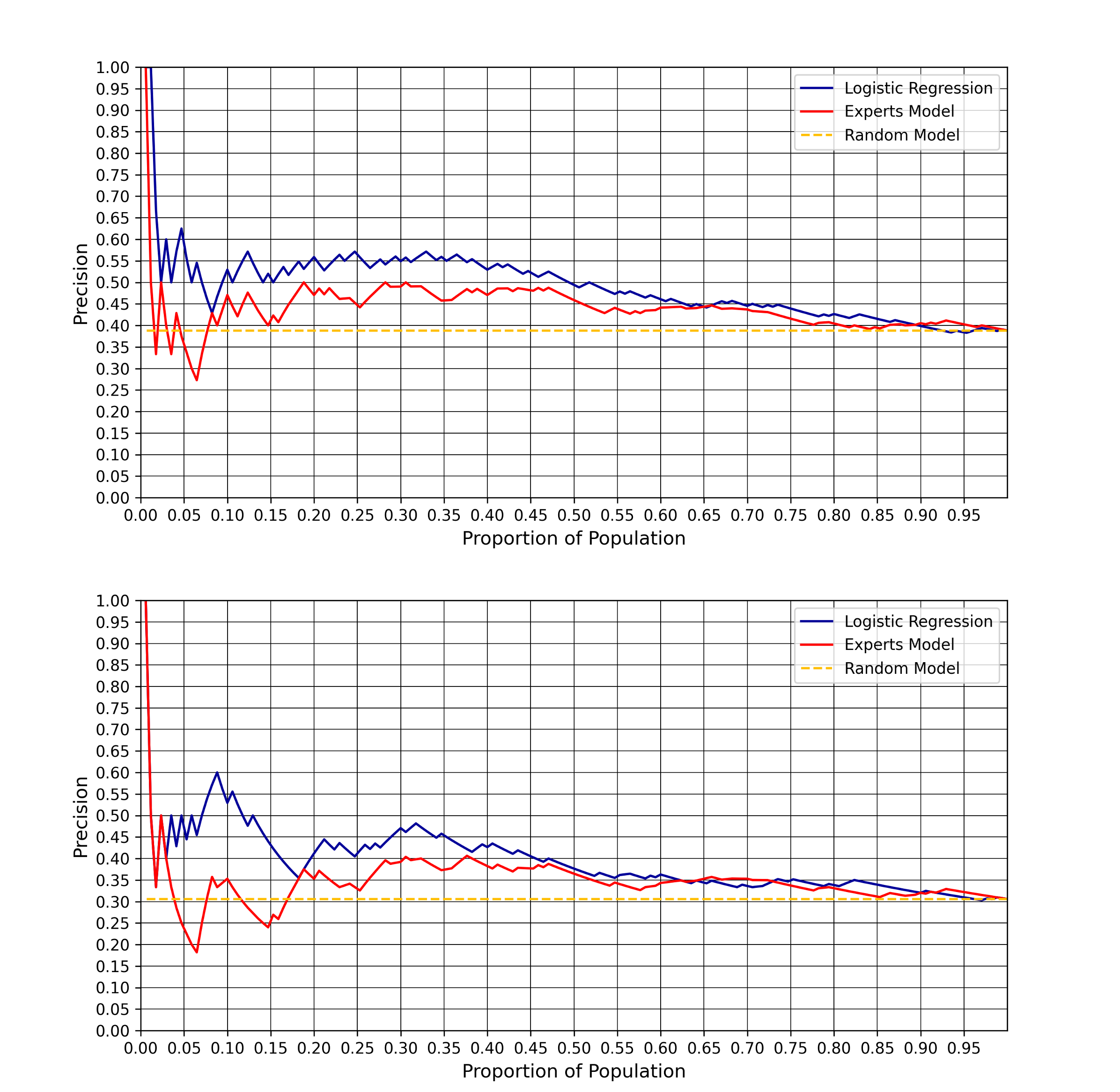


Figure S4.3. Precision curves for the DMFT3 model (above) and the DMFT4 model (below) comparing with both the experts and the random model, for the test subsample of students 12 or older, using DMFT (permanent teeth) as the target variable.
