# Supplementary material for: Low-cost and scalable machine learning model for identifying children and adolescents with poor oral health using survey data: An empirical study in Portugal
Source: PLoS One. 2025 Jan 24;20(1):e0312075. doi: 10.1371/journal.pone.0312075 (PMC11759376; doi:10.1371/journal.pone.0312075)
Supplement: S5 File — (DOCX) [file pone.0312075.s010.docx]

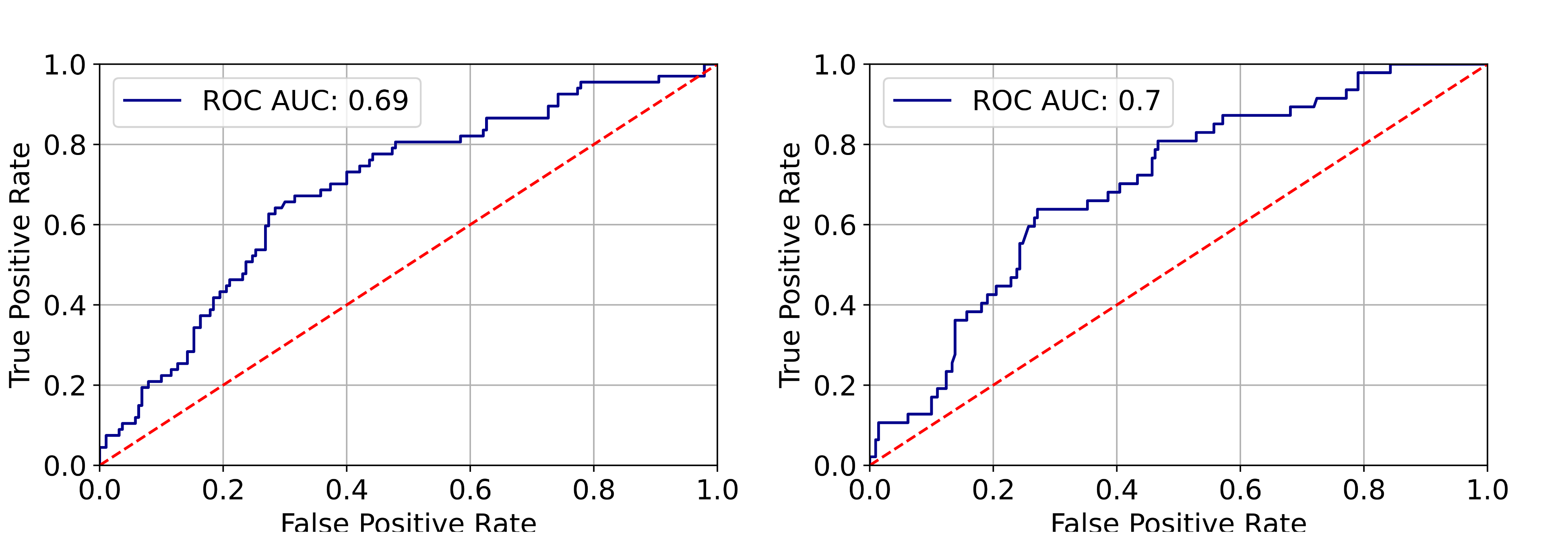


Figure S5.1. ROC curves for the DMFT3 model (left panel) and the DMFT4 model (right panel), for the test subsample of students younger than 12, using dmft (deciduous teeth) as the target variable.


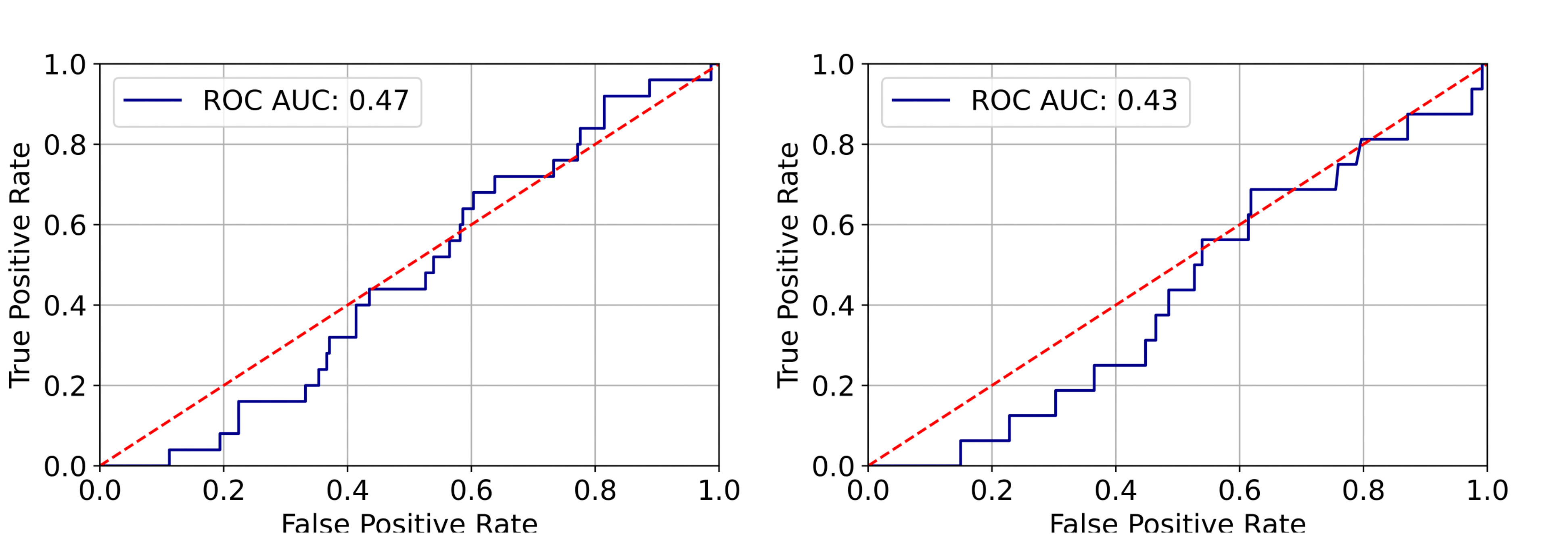


Figure S5.2. ROC curves for the DMFT3 model (left panel) and DMFT4 model (right panel), for the test subsample of students younger than 12, using DMFT (permanent teeth) as the target variable.


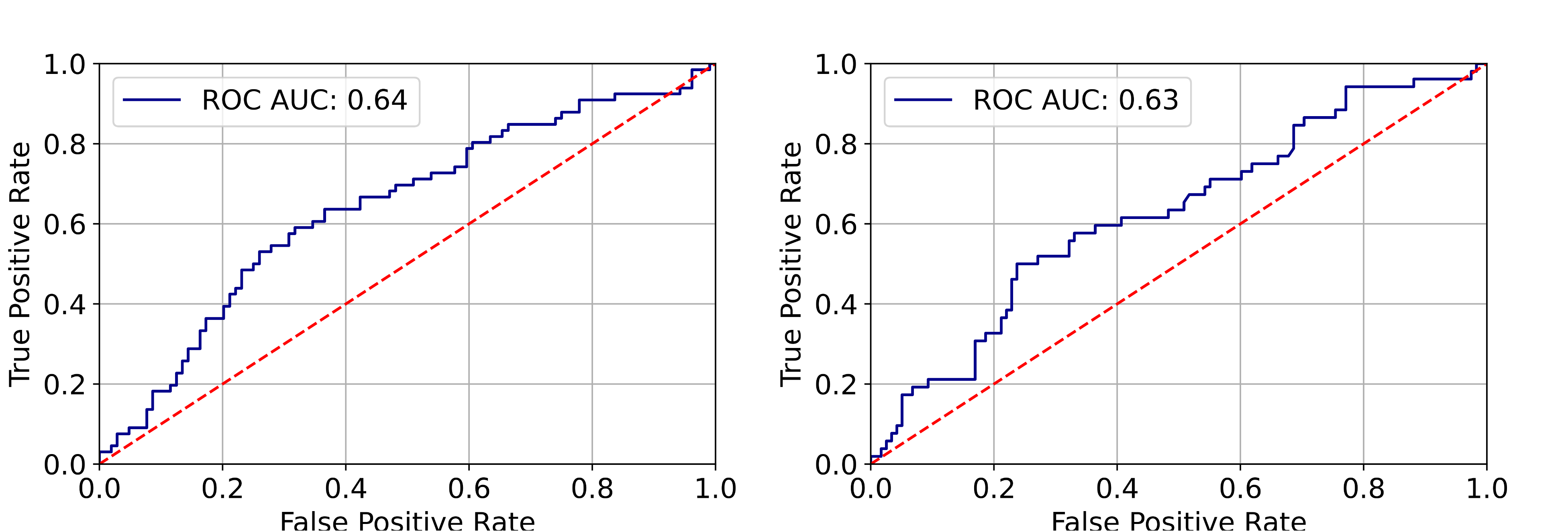


Figure S5.3. ROC curves for the DMFT3 model (left panel) and DMFT4 model (right panel), for the test subsample of students 12 or older, using DMFT (permanent teeth) as the target variable.
