# Supplementary material for: Low-cost and scalable machine learning model for identifying children and adolescents with poor oral health using survey data: An empirical study in Portugal
Source: PLoS One. 2025 Jan 24;20(1):e0312075. doi: 10.1371/journal.pone.0312075 (PMC11759376; doi:10.1371/journal.pone.0312075)
Supplement: S6 File — (DOCX) [file pone.0312075.s011.docx]

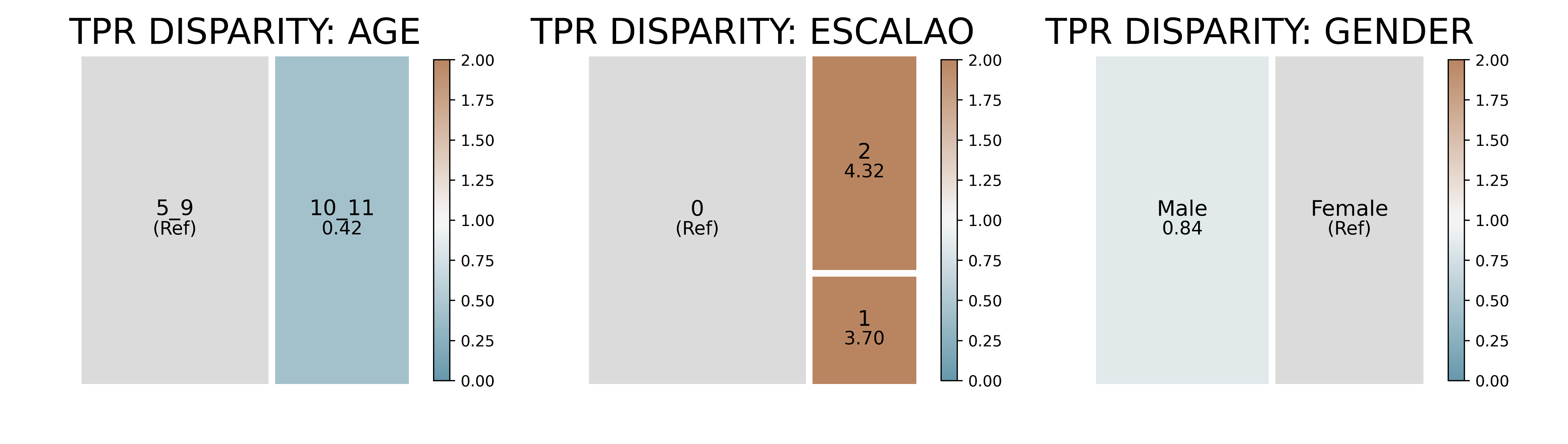
Figure S5.1. TPR disparity of the DMFT3 model when the 10% of the population with the highest model scores is selected as being at risk of poor oral health, for students younger than 12 when considering the dmft (deciduous teeth) as the target variable.


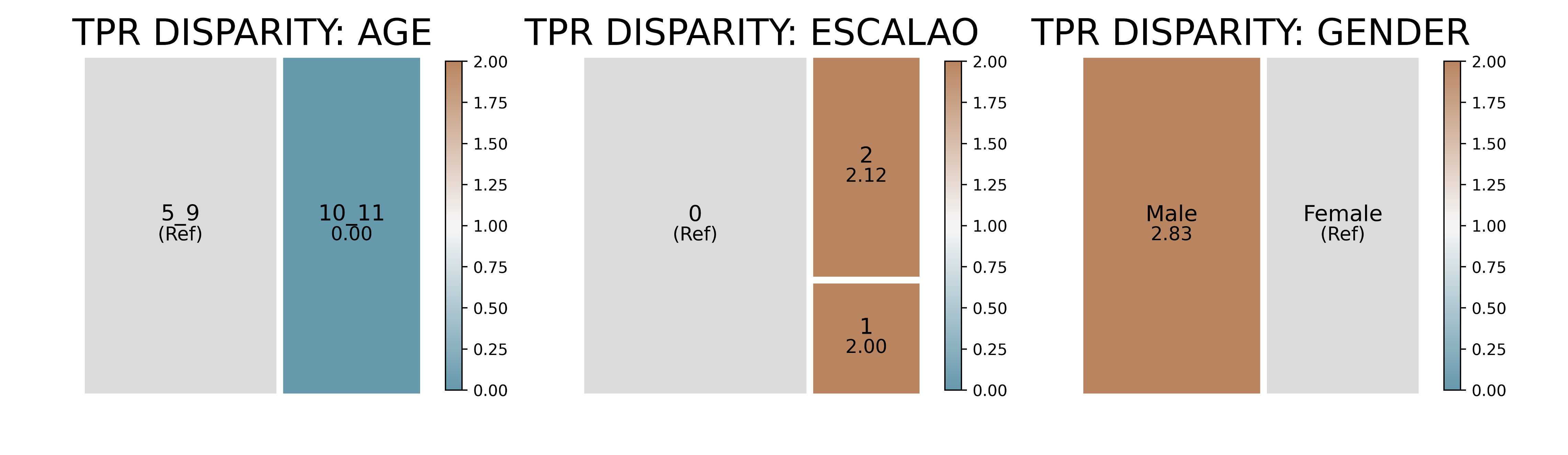
Figure S5.2. TPR disparity of the DMFT4 model when the 10% of the population with the highest model scores is selected as being at risk of poor oral health, for students younger than 12 when considering the dmft (deciduous teeth) as the target variable.


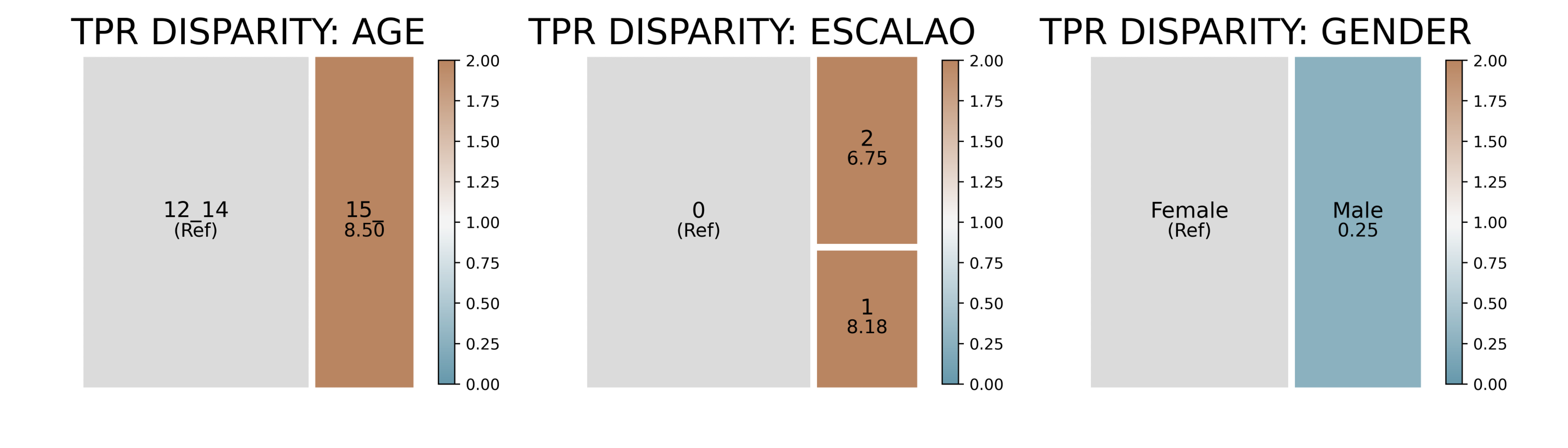
Figure S5.3. TPR disparity of the DMFT3 model when the 10% of the population with the highest model scores is selected as being at risk of poor oral health, for students 12 or older when considering the DMFT (permanent teeth) as the target variable.


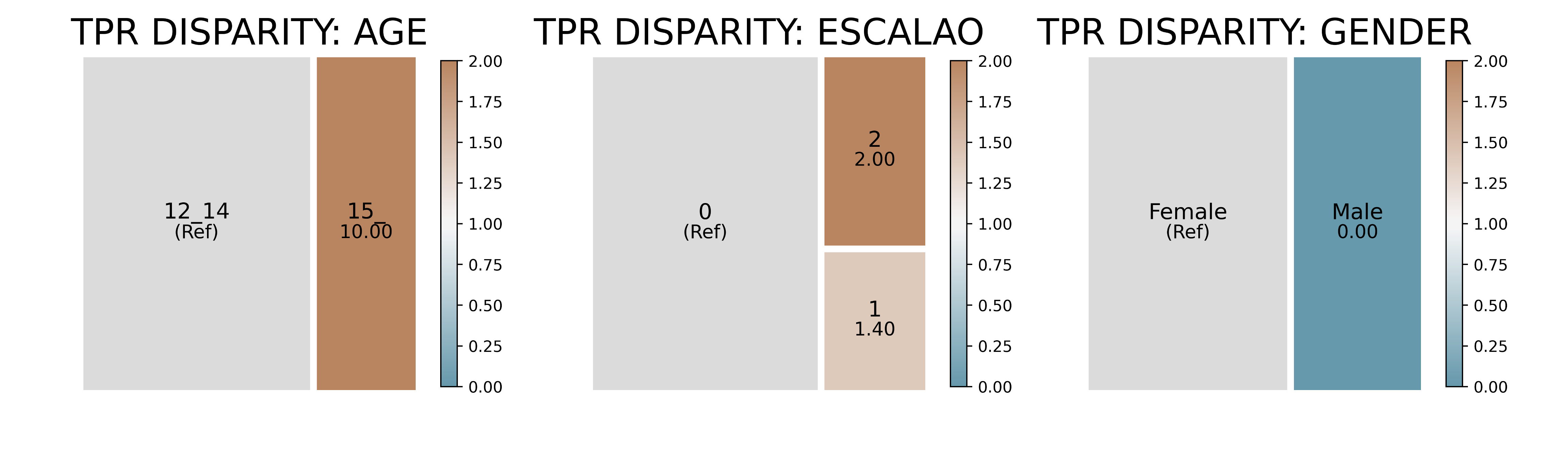
Figure S5.4. TPR disparity of the DMFT4 model when the 10% of the population with the highest model scores is selected as being at risk of poor oral health, for students 12 or older when considering the DMFT (permanent teeth) as the target variable.
